# Cascade Filtration With PCR Detection and Field-Flow-Fractionation Online With ICP-MS for the Characterization of DNA Interaction With Suspended Particulate Matter

**DOI:** 10.3389/fchem.2022.919442

**Published:** 2022-06-28

**Authors:** Volker Nischwitz, Lara Stelmaszyk, Sandra Piel, Andreas Tiehm

**Affiliations:** ^1^ Central Institute for Engineering, Electronics and Analytics, Analytics (ZEA-3), Forschungszentrum Juelich, Juelich, Germany; ^2^ Department Water Microbiology, TZW: DVGW Technologiezentrum Wasser, Karlsruhe, Germany

**Keywords:** antibiotic resistance genes, gene fragments, extracellular DNA, clay particles, cascade filtration, field-flow fractionation, DNA–clay adducts

## Abstract

The variety of applied antibiotics in animal and human medicine results in the release, development, and spread of relevant numbers of antibiotic resistance genes (ARGs) in the environment. The majority of ARGs are present in intracellular forms (in bacteria). Neglected aspects are extracellular variants of ARGs (eARGs) and their fragments, which have been detected in surface-water samples and sediments. The stability of eARGs is expected to be low; however, binding to particulate matter is likely to improve their stability and also affect their transport and dissemination behavior. Few studies have investigated DNA particle interactions, mostly *via* indirect characterization of adduct formation in model systems but not in real environmental matrices. Therefore, our study aims at a novel approach for direct characterization of desoxyribonucleic acid (DNA) particle interactions using both cascade filtration and field-flow fractionation. Cascade filtration with quantitative polymerase chain reaction (qPCR) detection indicated retention of ARGs on filters with much larger pore sizes supporting the hypothesis of ARG-particle interactions. However, artifacts from membrane clogging or DNA–membrane interaction cannot be excluded. Consequently, asymmetric flow field-flow fractionation was investigated as an alternative separation technique with the advantage of particle separation in a thin channel, reducing the risk of artifacts. The key method parameters, membrane composition, molecular weight cut off, and carrier composition, were systematically investigated using a calf-thymus DNA-spiked surface-water sample as a model. The results clearly showed a shift in the elution time of clay particles suggesting the presence of DNA–clay adducts. Multi-element detection by inductively coupled plasma mass spectrometry (ICP-MS) enabled monitoring of clay *via* the Al, Fe, and Si signals and DNA *via* the P signal. Matching peak profiles for the new fraction in the fractograms of the ARG and DNA-spiked water sample support adduct formation. Further evidence was provided by a novel post-channel filtration approach for the separation of free DNA from DNA–clay adducts.

## Highlights

• Cascade filtration for eARG particle aggregates with qPCR and ICP-MS detection.
• Optimization of FFF parameters for DNA–particle aggregates in the surface-water matrix.
• Detection of a new particle fraction in ARG and DNA-spiked water samples.
• Novel post-channel filtration approach for separation of free DNA and DNA–particle adducts.

## Introduction

Suspended particulate matter plays an important role in environmental surface water for the transport of bound nutrients and pollutants. The large structural variety of natural colloids in the nanometer to micrometer size range, including clays with or without organic surface coating by humic acids, offers multiple binding sites for interaction with dissolved species. Many studies investigated particle-bound phosphorus species as a significant contribution to nutrient cycles. Orthophosphate accounts for the far majority of dissolved and particle-surface-bound phosphorus amounts.

However, trace concentrations of phosphorus-containing (nano)pollutants, such as antibiotic resistance genes (ARGs) or their fragments, are critical health issues regarding water quality. The variety and large-scale application of antibiotics not only in human health care but also near industrial animal farming facilities cause an increased release of ARGs into the environment (Li et al., 2020; Stange and Tiehm, 2020; Voigt et al., 2020). Many studies reported the detection of ARGs in surface waters (e.g., Stoll et al., 2012; Exner et al., 2018; Stange et al., 2019; Chaturvedi et al., 2021) and sediments (Calero-Cáceres et al., 2017), and advanced membrane filtration devices were developed to eliminate ARGs during water purification (Niestroj-Pahl et al., 2020). Challenges are the occurrence of ARGs not only in bacteria but also as extracellular bound or free forms (eARG) (Nielsen et al., 2007; Ibáñez de Aldecoa et al., 2017). Their occurrence in small ARG-carrying fractions, such as environmental bacteriophages, has already been shown to contribute significantly to their dissemination (Calero-Cáceres et al., 2014). According to a study by Dong et al. (2019), the adsorption of extracellular ARGs on the surface of particulate matter is also an important aspect to understand their transport, stability, and also transforming ability. In this study, high amounts of eARGs (next to intracellular ARGs) were detected in urban lake sediments, indicating the relevance of those extracellular genes for the spread of resistance in the aquatic environment. Incubation experiments of DNA with particulate matter have been performed for model systems, but systematic investigations of such interactions are rare for real natural water matrices. Pietramellara et al. (2007) compared the adsorption of pure DNA and dirty DNA containing, i.e., cellular wall debris with clay minerals and found facilitated adsorption for the dirty DNA. The adsorbed amount of DNA was determined indirectly based on DNA measurement in the supernatant after centrifugation. After several washing steps of the DNA–clay-containing pellet, the calculated amount of bound DNA was accounting for only about 10% of the adsorbed DNA. The supporting effect of amino acid buffers was reported for the adsorption of DNA to silica surfaces (Vandeventer et al., 2013). Griffith et al. (2020) concluded the formation of a DNA corona in analogy to a protein corona when incubating 200 nm and 1 µm polystyrene nanoparticles with calf-thymus DNA in phosphate-buffered saline at pH 7.3–7.5. DNA adsorption on cationic particles was about three times higher than on anionic particles.

Recently, Chowdhury et al. (2021) reported batch experiments of extracellular ARGs and 100 nm nominal-sized silica nanoparticles (hydrodynamic diameter 135.60 ± 1.29 nm) or micron-sized kaolinite (hydrodynamic diameter 865.73 ± 59.07 nm) in presence of humic acid and divalent cations (CaCl_2_) at pH 7. Adsorption isotherms were established based on fluorimetric measurement of the free ARG concentration in solution. These results indicated ARG adsorption to silica particles in presence of 10 mM CaCl_2_ or 20 mg L^−1^ humic acid previously bound to the silica surface. Complementary Fourier transform infrared (FTIR) measurements demonstrate slight changes in the spectra for ARGs incubated with humic-acid-modified silica particles and clearer changes in the case of ARGs incubated with kaolinite. Washing with Tris buffer released about 17–45% of bound ARGs. These model experiments demonstrate the interaction of ARGs with particles in presence of supporting buffers. However, the binding affinity and stability of the formed aggregates need to be studied in more detail under real environmental surface water matrix conditions.

Apart from the FTIR results by Chowdhury et al. (2021), the discussed studies are based on indirect characterization of ARG-particle interaction *via* the difference between the spiked amount of DNA and the free amount of DNA in solution. Separation of the ARG-binding particles from the original particles and the free/desorbed ARGs has the potential for more reliable proof of binding and a more detailed characterization of the stability of the interaction. Particle size fractionation could either be achieved by a cascade filtration (CF) with decreasing filter pore sizes or by asymmetric flow field-flow fractionation (AF4). The experimental setup of CF is easier and fractionation faster compared to AF4. For example, Riquelme Breazeal et al. (2013) performed cascade microfiltration and ultrafiltration steps, including 0.45 µm, 0.1 µm, 100 kDa, 10 kDa, and 1 kDa for ARG-spiked effluent from waste water treatment plants. Significant removal of ARGs was achieved for membranes with a nominal pore size of 100 kDa and smaller. The particle-bound ARG fractions can be recovered from the 0.45 and 0.1 µm membranes, while the free ARGs are mainly expected on the 100 kDa membrane. Some limitations may occur due to partial clogging of the filters or adsorption of DNA on the membranes. In addition, it needs to be considered that the nominal pore size of the filters is typically not identical to the smallest retained particle size (Yang et al., 2020). The CF fractions are available for offline detection.

AF4 requires a higher instrumental setup but has the advantage of a continuous particle separation profile in a thin channel without the need for a stationary phase. Interaction with the membrane needs to be minimized by suitable carrier composition and membrane selection. Detection can be performed either online or offline via collection of fractions (Meermann and Nischwitz, 2018). Few AF4 applications for the characterization of DNA have been published (Kim et al., 2012). Initial studies focused on the separation of various forms and molecular sizes of DNA using 0.1 mol L^−1^ Tris buffer with 1 mmol L^−1^ EDTA at pH 8 (Liu and Giddings, 1993). Cationic lipid–DNA complexes were characterized using either deionized water with 0.02% sodium azide as a carrier or 0.089 mol L^−1^ Tris-borate buffer at pH 8.59 (Lee et al., 2001). A comparison of three membranes indicated the best recoveries for a polypropylene membrane. The method optimization aimed at minimum interaction between the lipid–DNA complex and the separation system to maintain the complex stability during separation. Ashby et al. (2014) demonstrated that AF4 with UV/VIS and fluorescence detection is an effective technique for studying DNA–protein interactions using a regenerated cellulose membrane with phosphate-buffered saline as a carrier. The application of AF4 for the characterization of DNA adducts with environmental particles has not been reported yet.

Selective ARG detection in water samples is routinely performed by PCR after suitable pre-concentration (Stange et al., 2019). This sensitive approach requires significant effort for sample preparation, rather large sample volume (>100 mL), and cannot be performed online with a separation system. Matrix components, such as humic acids or organic-acid-based buffers, cause interferences in PCR detection. Less selective but more flexible and robust is the detection of ARGs with inductively coupled plasma mass spectrometry (ICP-MS) *via* the phosphorus signal, as previously demonstrated for other DNA applications (Cuello-Nuñez et al., 2017).

The discussed results from published studies highlight the need for a more detailed characterization of ARG-adducts with environmental nanoparticles and colloids in a real surface-water matrix. Initial experiments in our study were performed by cascade filtration with qPCR quantification. As a novel approach, AF4 online with ICP-MS was subsequently applied. This method has the potential for the separation of suspended particulate matter and the corresponding ARG-adducts combined with online monitoring of ARG fragments *via* the phosphorus signal and of the particles *via* their core elements, including iron (Fe) and aluminum (Al). Challenges are the selection of suitable membrane material and molecular weight cut off for the field-flow fractionation (FFF) channel and a carrier composition as the best compromise between stability of the DNA adducts, compatibility with the environmental sample matrix, separation efficiency, and recovery. Due to the limited amount available of the produced ARG fragments, calf-thymus DNA was used to prepare spiked model water samples for FFF-ICP-MS method development. The optimized method was applied to ARG-spiked water samples to verify the results obtained with the model sample.

## Materials and Methods

### Cascade Filtration

For the cascade filtration (CF), four filters were used for sequential filtration of a water sample, namely, 5 and 1.2 µm pore size filters (polycarbonate (PC), Merck Millipore) and polyethersulfone (PES)-membranes of 0.45 µm und 0.2 µm pore size (Supor, PALL). Filtrates were collected after each vacuum filtration in a clean and sterilized glass bottle and used for further filtration. After cascade filtration, the filtrate was concentrated by stirred cell ultrafiltration (Amicon Stir Cell 400, Merck Millipore) using a 5 kDa MWCO PES membrane (Merck Millipore), which was previously incubated with a 250 mM AlCl_3_ solution (aluminum chloride hexahydrate, Merck Millipore) for 2 min for better retention of eDNA (Haramoto et al., 2005). The pre-concentration factors of each filtration and pre-concentration step were considered for the calculation of the gene copy concentration per mL of the original sample.

#### Surface-Water Sampling

Two surface-water samples were collected in May and August 2021 from river Alz, Emmerting, Bavaria, Germany. Physico-chemical parameters were partly determined and are provided in Supplementary Information (Supplementary Table S2). The samples were stored at 4°C before use and were processed after 24 h or less.

#### ARG Analysis

Previously to the qPCR reaction, the total DNA was extracted directly from the membranes by using the FastDNA™ SPIN Kit for soil (MP Biomedicals) according to the manufacturer’s instructions with a final elution volume of 100 μL. For the investigation of gene copies in the filtrates, 1 µL of the filtrates was directly used as a template for the qPCR reaction. The previously published primer sets and qPCR conditions, as shown in Table 1, were used. All qPCRs were performed using a Rotor-Gene 6000 cycler (Corbett) with SsoAdvanced Universal SYBR Green Mix (Bio-Rad). The temperature profile for the amplification was as follows: 2 min 98°C (initial phase for enzyme activation), 45 cycles of 20 s at 98°C (denaturation), 20 s at primer specific annealing temperature (T_A_), and fragment-length-dependent elongation time (t_E_) at 72°C, followed by melting curve analysis.

**TABLE 1:** Primers and qPCR conditions of the genes studied (TA, Annealing Temperature; tE, Elongation time).

Gene	Amp. [bp]	Forward Primer (5′-3′)	Reverse Primer (5′-3′)	TA [°C], t_E_ [s]	Ref.
*16S rDNA*	160	CCTACGGGAGGCAGCAG	ATTACCGCGGCTGCTGGC	68, 20	Muyzer et al. (1993)
*intl1*	196	GCC​TTG​ATG​TTA​CCC​GAG​AG	GATCGGTCGAATGCGTGT	63, 20	Barraud et al. (2010)
*sul1*	163	CGC​ACC​GGA​AAC​ATC​GCT​GCA​C	TGA​AGT​TCC​GCC​GCA​AGG​CTC​G	68, 20	Pei et al. (2006)
*sul2*	722	CGG​CAT​CGT​CAA​CAT​AAC​C	GTGTGCGGATGAAGTCAG	65, 25	Maynard et al. (2003)
*blaCMY-2*	172	CGT​TAA​TCG​CAC​CAT​CAC​C	CGT​CTT​ACT​AAC​CGA​TCC​TAG​C	68, 20	Kurpiel and Hanson, (2011)
*blaTEM*	112	TTC​CTG​TTT​TTG​CTC​ACC​CAG	CTC​AAG​GAT​CTT​ACC​GCT​GTT​G	66, 20	Bibbal et al. (2007)
*blaCTXM-32*	155	CGT​CAC​GCT​GTT​GTT​AGG​AA	CGC​TCA​TCA​GCA​CGA​TAA​AG	63, 20	Stalder et al. (2014)
*blaSHV*	857	TCG​CCT​GTG​TAT​TAT​CTC​CC	CGC​AGA​TAA​ATC​ACC​ACA​ATG	55, 30	Maynard et al. (2003)
*blaNDM-1*	154	ATTAGCCGCTGCATTGAT	CAT​GTC​GAG​ATA​GGA​AGT​G	60, 20	Naas et al. (2011)
*blaOXA-48*	177	TGT​TTT​TGG​TGG​CAT​CGA​T	GTAAMRATGCTTGGTTCGC	55, 20	Monteiro et al. (2012)
*blaVIM-2*	382	GTT​TGG​TCG​CAT​ATC​GCA​AC	AATGCGCAGCACCAG GATAG	63, 20	Li et al. (2015)
*vanA*	1030	CAT​GAA​TAG​AAT​AAA​AGT​TGC​AAT​A	CCC​CTT​TAA​CGC​TAA​TAC​GAT​CAA	60, 35	Clark et al. (1993)
*mcr-1*	183	GGG​CCT​GCG​TAT​TTT​AAG​CG	CAT​AGG​CAT​TGC​TGT​GCG​TC	68, 20	Hembach et al. (2017)
*mecA*	91	CGC​AAC​GTT​CAA​TTT​AAT​TTT​GTT​AA	TGG​TCT​TTC​TGC​ATT​CCT​GGA	63, 20	Volkmann et al. (2004)

All samples and standards were analyzed in duplicates. The qPCR standards were prepared by serial dilutions of known quantities of a linearized plasmid containing target genes. For quality control, *R* ^2^ of the standard curve and the amplification efficiency were determined, and melt-curve analysis was performed. Only qPCR experiments with *R* ^2^ values > 0.990 and efficiencies between 90 and 105% were considered. Amplification products were verified via the QIAxcel^®^ Advanced system (Qiagen). An overall limit of quantification was 10 copies per qPCR reaction.

The same PCR conditions were also applied to a standard fragment of the ARGs as a template, to produce the fragments for model experiments. After their production with PCR, they were purified with the high pure PCR product purification kit (Roche Diagnostics), and DNA concentration was measured with a Qubit™ nanophotometer with the ds DNA BR assay kit (Life Technology).

#### Preparation of Model Water Samples for Cascade Filtration

Silica particles (see *Chemicals and Reagents Section*) were incubated with ARG fragments in autoclaved and deionized water (Milli-Q), in autoclaved drinking water (3.2 mM CaCO_3_, Karlsruhe, Hagsfeld), and in sterile-filtered amino acid buffer (0.1 mol L^−1^ Arginin, CELLPURE^®^ ≥99%, Carl Roth in 0.4 mol L^−1^ KCl, min. 99.5%, for cell culture, Carl Roth, pH 5) or acetic acid buffer (0.25 mol L^−1^ acetic acid, 99.8% Carl Roth in 0.4 mol L^−1^ KCl, pH 5). For comparison, incubations without silica particles were prepared in analogy. More specifically, the following components were used: eDNA-fragment intl1 (196 bp) at 1 mg L^−1^, eDNA-fragment sul2 (722 bp) at 1 mg L^−1^, and Sicastar silica-particles (nominal size 100 and 500 nm at 1 g L^−1^ each). The suspensions were subjected to sequential filtration using filters described in the *Cascade Filtration Section*. The model water samples and filtrates were analyzed by qPCR and quadrupole ICP-MS for total phosphorus and silicon using He collision cell mode (Agilent 7500, Agilent Technologies, Japan) monitoring ^13^C, ^31^P, ^28^Si, ^29^Si, and ^103^Rh. External calibration was performed for P in the range from 1 μg L^−1^–4000 μg L^−1^ and Si in the range from 10 μg L^−1^–40,000 μg L^−1^ with 10 μg L^−1^ Rh as the internal standard. Commercial NIST traceable calibration stock solutions were used for P (Merck, Darmstadt, Germany), Si (VWR International, PA, USA), and Rh (Alfa Aesar, Kandel, Germany). Samples were analyzed at 5 to 20 fold dilution. ^13^C was monitored to check for carbon-based interferences on Si-quantification, in particular, for the amino acid and acetic-acid-buffered fractions. For all solvents, control experiments without silica particles were performed to check for blank levels of dissolved silicon in the solvents and potential interferences: <0.06–0.18 mg L^−1^ for deionized water, 6.1–6.9 mg L^−1^ for drinking water, 1.0–1.2 mg L^−1^ for an acetic acid buffer, and 2.1–2.3 mg L^−1^ for amino acid buffer. These concentrations were negligible compared to the feed Si concentration of about 400 mg L^−1^ in the CF experiments with silica particles. Digestion of the particles was not required, as demonstrated by Prestel et al. (2005), showing no significant difference in ICP-MS response for silica particles in the range from 100 to 500 nm, which was confirmed by our experiments using flow injection for quantification of the 100 and 500 nm silica particles used in this study (Supplementary Figure S2).

### Asymmetric Flow Field-Flow-Fractionation Online With ICP-MS

#### Chemicals and Reagents

Sodium chloride (EMSURE, p.a.) and hydrochloric acid (suprapure) were purchased from Merck, Darmstadt, Germany. Tris buffer and calf-thymus (CT) DNA sodium salt were obtained from Alfa Aesar, Kandel, Germany and ammonium acetate (>99%) from Honeywell, Seelze, Germany. Plain silica particles Sicastar (nominal size 100 and 500 nm, both at a concentration of 50 g L^−1^) in aqueous suspension were supplied by MicroMod, Rostock, Germany. The zeta potential of these particles is −5.8 mV at pH 4.4, −28.7 mV at pH 5.8, and −37.2 mV at pH 7.5, according to the manufacturer, and the determined hydrodynamic diameter is 109.3 and 527.8 nm, respectively.

#### Sampling

A surface-water sample (Hbh1) was collected within an agricultural area in northern Baden-Württemberg, Germany in July 2021. The sample was stored at 4°C and filtered through a 5 µm membrane before use to exclude particles above the separation range of AF4. The pH was 8.0 and the conductivity was 292 μS cm^−1^.

#### Sample Preparation

The model sample for FFF-ICP-MS method development was regularly prepared by spiking CT-DNA into the surface-water sample Hbh1 and used for a maximum of 2 days. More specifically, the CT-DNA stock solution in deionized water (4 g L^−1^) was diluted with the surface-water sample Hbh1 up to 1000 fold. Higher spike levels (dilution 500-fold and 333-fold) were once prepared for comparison. ARG fragments (*sul2*, 722 bp, 530 kDa, ca. 30 mg L^−1^ and *mecA*, 91 bp, 56 kDa, ca. 5 mg L^−1^) were prepared *via* PCR amplification with the primers listed in Table 1 and spiked into the water sample Hbh1 at 20-fold and 4-fold dilution, respectively.

#### Instrumental Setup

An AF 2000 system from Postnova, Landsberg, Germany was used with an analytical FFF channel. The spacer thickness was 500 µm and the detector flow was 0.5 mL min^−1^. The injection volume of the samples was 0.5 mL or 1 mL with a focusing time of 10 min or 2 mL with a focusing time of 20 min. The AF4 was coupled to a quadrupole ICP-MS Agilent 7500 (Agilent Technologies, Japan) *via* a PEEK transfer line, including a T-piece for the introduction of an internal standard solution (10 μg L^−1^ Rh in 0.5% hydrochloric acid). The ICP-MS was equipped with a MicroMist nebulizer and double-pass spray chamber and operated in He collision cell mode (plasma power 1500 W; He flow rate 4 mL min^−1^; quadrupole bias −16 V; and octopole bias −18 V for kinetic energy discrimination) for monitoring of ^27^Al, ^28^Si, ^31^P, ^56^Fe, and ^57^Fe and ^103^Rh as the internal standard. The priority was on accurate quantification of the low phosphorus concentrations from DNA and therefore He-mode was selected as an optimum condition. Quantification of the eluting elemental fractions was performed by a post-channel calibration approach, as described in previous work by Nischwitz and Goenaga-Infante (2012). Briefly, calibration standards in the concentration range of 25 μg L^−1^ to 1000 μg L^−1^ for P and in the range of 250 μg L^−1^ to 10,000 μg L^−1^ for Al, Si, and Fe (containing 10 μg L^−1^ Rh in 0.5% hydrochloric acid) were introduced via the T-piece during a blank run of the FFF to establish a calibration curve. Based on this calibration, the raw data were converted into mass-flow fractograms and the absolute elemental mass per fraction was determined by peak integration. The concentration of the particulate elemental fractions was calculated as a ratio to the injection volume.

#### Optimization of the Cross Flow

Based on our previous work (Yang et al., 2020), the cross flow was varied from 2 mL min^−1^ to 1 mL min^−1^. As an alternative to the power cross-flow gradient also a linear gradient was explored. The final method started with a constant cross flow of 1 mL min^−1^ for 22 min followed by a power gradient down to zero within 49 min and finally 40 min at zero cross flow.

#### Selection of the Membrane and the Carrier

Several membranes were compared for their performance: 1 kDa PES, 1 kDa regenerated cellulose (RC), 50 kDa polyvinylidene fluoride (PVDF), and 150 kDa PVDF. All membranes were tested with a 25 μmol L^−1^ NaCl aqueous carrier. Alternatively, a carrier composition of 5 mmol L^−1^ ammonium acetate, pH 7 was used with the 1 kDa PES membrane and a carrier of 5 mmol L^−1^ Tris buffer at pH 5 with the 50 kDa PVDF membrane.

#### Post-Channel Filtration

A 100 nm membrane was inserted into an in-line filter cartridge and placed in the transfer line between the FFF channel and the ICP-MS to separate clay particles and their DNA adducts from the smaller unbound DNA. The filter membrane was changed after each run.

#### Data Processing

The fractograms are presented after normalization to the internal standard Rh. In addition, five-point average smoothing was applied.

## Results and Discussion

### Cascade Filtration of a Surface-Water Sample

**FIGURE 1:**
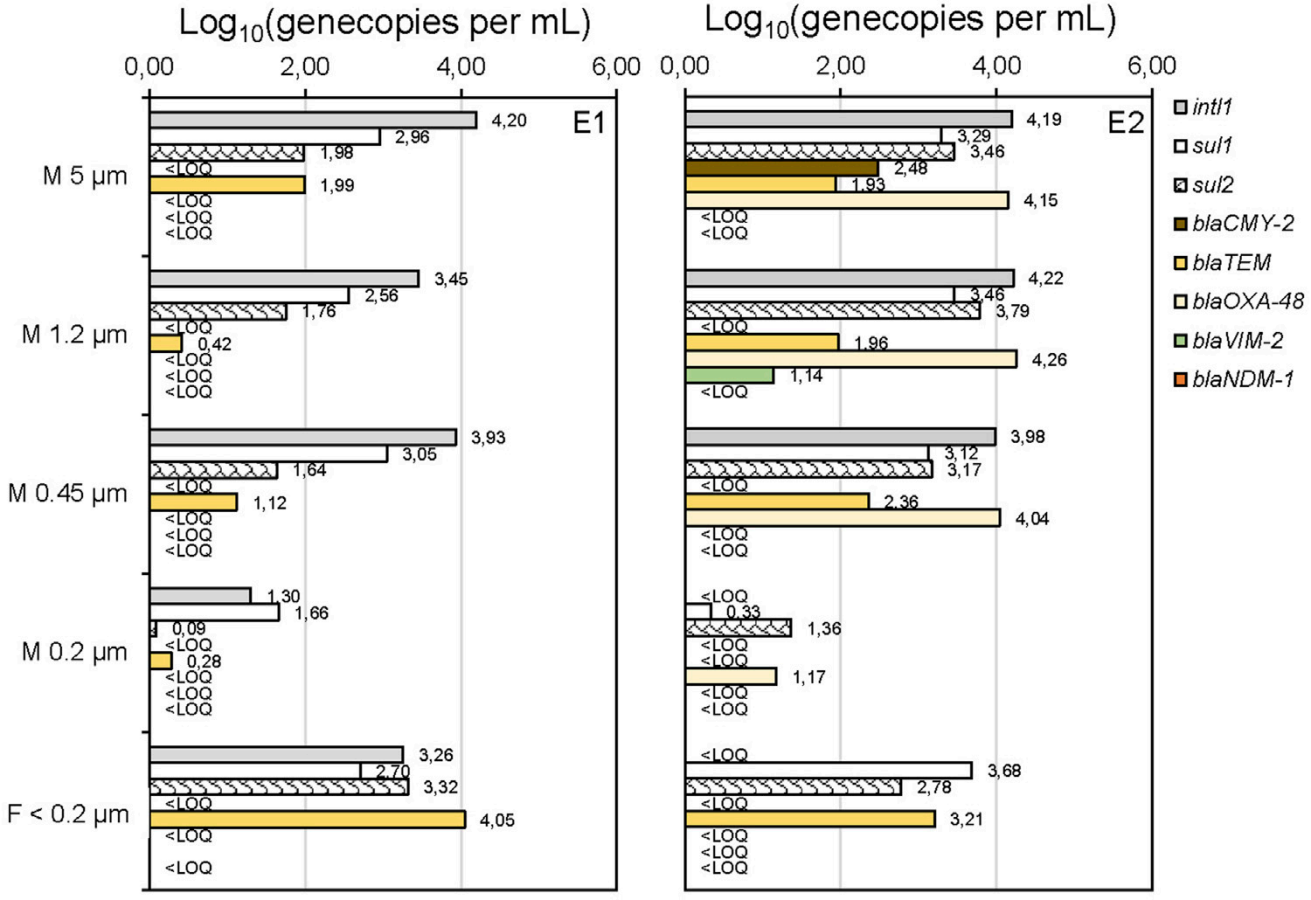
Application of cascade filtration on surface-water samples (river Alz, Germany, May (E1) and August (E2) 2021) and analysis of gene copies (GC)/mL after DNA extraction from the membrane (M 5–0.2 µm) and without DNA extraction in the filtrate <0.2 µm. Limit of quantification (LOQ) was about 1 gene copy per mL.

Surface-water samples from river Alz (May and August 2021, E1 and E2) were examined for ARGs after cascade filtration (CF) and for comparison after using the conventional treatment method (filtration through a 0.2 µm membrane only). The sum of the gene copies quantified after each step of the CF was very similar to the GC concentrations obtained by the standard filtration (data not shown, see Supplementary Figure S1). In Figure 1, the investigated sulfonamide resistance genes *sul1 and sul2;* the *β*-lactamase genes *blaCMY-2, blaTEM, blaOXA-48, blaVIM-2*, and *blaNDM-1*; and the integrase gene *intl1* are shown according to the retention on the applied filters.

Other resistance genes of different antibiotic classes, including *vanA*, *blaSHV*, *mecA*, and *mcr-1*, were examined, but could not be detected in these samples. The retentate on the membranes 5–0.45 µm was expected to be representative of antibiotic-resistant bacteria and ARGs associated with larger-sized sediment particles. The fact that these fractions can strongly adsorb to sediment particles can be explained by the predominantly positive surface charge of the particles and the negative charge of these biomolecules and is also confirmed by the literature (Mao et al., 2014; Torti et al., 2015; Sivalingam et al., 2020; Zarei-Baygi and Smith, 2021). Compared to the gene concentrations observed on the 0.2 µm membrane, all detected genes are present at significantly higher concentrations on the membranes of 5 μm, 1.2 μm, and 0.45 µm. The integrase gene *intl1,* which is mainly associated with bacterial mobile genetic elements and chromosomal DNA (Mazel, 2006), is constantly detectable in these fractions with concentrations of 10^3.5^–10^4.3^ GC mL^−1^, suggesting intracellular ARGs to be the predominant fraction. This hypothesis can be supported by the reduced *intl1* concentrations of only 10^1.3^ GC mL^−1^ or below the limit of quantification (LOQ) on the 0.2 µm membranes, where most bacteria are expected to be retained as their typical sizes range from 0.4 to 3 µm (Levin and Angert, 2015). Also concentrations of *sul1,* which are reduced about 10-fold compared to *intl1*, are significantly increased on membranes 5–0.45 µm (E1 and E2). This result fits with a study showing that integrons are frequently associated with sulfonamide resistances (Chaturvedi et al., 2021). The occurrence of some *β*-lactamase genes, namely, *blaCMY-*2, *blaOXA-48,* and *blaVIM-2* in the fractions ≥0.45 µm in only one of the samples might be explained by higher inputs of wastewaters upstream the river in August, when heavy rainfalls might have led to stormwater overflow in waste water treatment plants. Free DNA (eDNA) is expected to pass through the 0.2 µm membrane. Therefore, the ARGs detected on the 0.2 µm membrane are likely to represent ARGs adsorbed to nanoparticles or colloids. Experiments conducted by Chowdhury et al. (2021) showed that this sorption of eARGs to nanoparticle surfaces is irreversible under specific conditions (humic-acid-functionalized silica nanoparticles) and to protects ARGs from DNAse I degradation (Chowdhury and Wiesner, 2021). Moreover, DNA was found to adsorb better in sand and mineral surfaces, in general, under low pH conditions and higher chaotropic salt concentrations (Lorenz and Wackernagel, 1987; Romanowski et al., 1991). Therefore, the adsorption of eARGs to particulate matter is seen as an important parameter for the investigation of ARG persistence in the aquatic environment. In the filtrate <0.2 µm the gene copy concentrations for *sul1* and *sul2*, as well as *blaTEM,* are again significantly increasing in both samples. From these results, an occurrence of ARGs in the fractions of eDNA (desorbed from particles) could be assumed. In case of the filtrate <0.2 µm, the pre-concentration factor was lower than for the previous CF steps, which may affect the direct comparability of the gene copy concentrations. However, the important result is that ARGs could be detected in the filtrate <0.2 µm, which was only possible after pre-concentration. This emphasizes the importance of further processing the filtrate to improve limits of detection and to avoid an underestimation of ARGs in aquatic samples. Similar findings have been already published for the analysis of eARGs in urban lake sediments, which were reservoirs of ARGs like *blaTEM* and *sul1* and *sul2* (Dong et al., 2019).

Although differentiation of ARG concentrations among the different fractions was obtained with CF, the ARGs association to different particle sizes cannot be proven with the CF separation method and therefore must be verified by additional methods and experiments. The detection in the separate fractions cannot be seen as proof of associations to different-sized particles. Critical issues and limitations are the unknown composition of the sample, potential membrane fouling, and the possibility of cell disruption during CF. Therefore, complementary analytical techniques are required to confirm the association of genes with different-sized particle fractions potentially indicated by CF with subsequent qPCR analysis of the ARGs.

### Investigation of ARG Binding to Silica Particles by Cascade Filtration

The same cascade filtration setup was applied to model water samples containing known concentrations of silica nanoparticles and gene fragments representing eARGs to investigate the separation capability and the limitations experienced with the surface-water sample in more detail. A variety of buffer systems is reported in the literature to support the binding of DNA to particles. However, most of the buffers are not compatible with the alternative analytical approach used in this study (AF4 online with ICP-MS) due to high salt concentrations causing clogging of the cones in the ICP-MS interface. Therefore, CF with offline ICP-MS and qPCR detection was performed to compare amino acid buffer (AmA), acetic acid buffer (AcA) (Vandeventer et al., 2013), drinking water (D), and ultrapure deionized water (U) regarding their efficiency in supporting ARG-representative gene fragments binding to silica particles of 100 and 500 nm. The silica particles were selected as model for natural mineral particles. Cascade membrane filtration was applied for particle size fractionation to determine the amount of genes retained with the silica particles. Since silicon (Si) and phosphorus (P) measurements with ICP-MS were only possible for the filtrates, not for the membrane retentates, ARG fragments were also determined in the filtrates by qPCR for better comparison of DNA and particles in the various size fractions.

**FIGURE 2:**
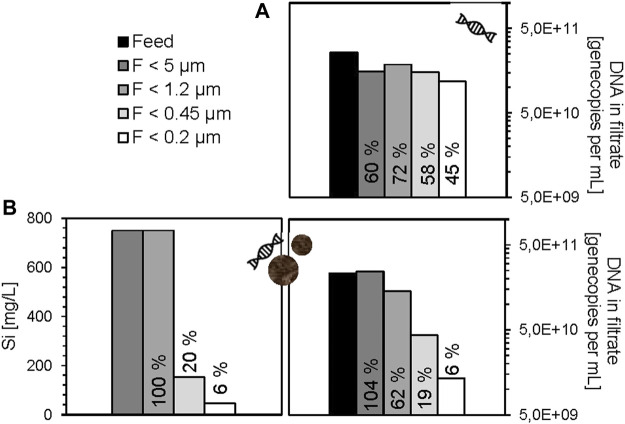
Free DNA (eARG-fragment *sul2* and eDNA fragment *intl1*) in filtrates of cascade filtration experiments without silica particles **(A)** and with silica particles **(B)** in ultrapure water. The percentage permeation of each step was calculated from the concentration of gene copies per mL filtrate divided by gene copies per mL feed (C_fil_/C_feed_ *100). Si concentration is shown for the filtrates of the experiment with DNA and silica particles; in absence of silica particles, the Si concentration was at a blank level <0.3 mg L^−1^.

For the silica particles, it was expected that the 500 nm size, which means 50% of total silicon, would be retained by the 0.45 µm membrane, and the 100 nm size, which means also 50% of total silicon, would not be retained. In contrast to that, ICP-MS measurements revealed in deionized water a retention of 80% of the total silicon on the 0.45 µm membrane, while the retention was even higher for amino acid buffer (97%), drinking water (98%), and acetic acid buffer (99%). The much-higher retention rate might be due to rapid membrane fouling or clogging by the larger silica particles causing co-retention of smaller particles. In addition, aggregate formation of the silica particles by surface interaction with DNA fragments and cations present in the drinking water is likely. Complementary qPCR measurements of the free DNA fragments in the filtrates were performed for ultrapure water and drinking water. The experiment with eDNA fragments and silica particles of sizes 100 and 500 nm resulted in only approximately 6% of the eDNA fragments in the filtrate <0.2 µm, while 19% passed through the 0.45 µm membrane and 62% through 1.2 µm membrane (Figure 2). In comparison, the same filtration experiment with eDNA fragments in the absence of silica particles showed a permeation of about 60–70% through 5 μm, 1.2 and 0.45 µm membranes followed by slight decrease down to 45% through the 0.2 µm membrane. In theory, the DNA molecule sizes are not exceeding 530 kDa (∼max. 5 nm) and therefore should pass through the whole cascade. The partial retention of DNA during filtration in absence of silicon might be due to an interaction with the filtration device or the 5 µm membrane under neutral pH and salt-free conditions or due to partial aggregation or instability of DNA under these conditions. However, as the permeation through 0.45 and 0.2 µm membrane is still much higher, compared to the experiment with silica particles and ARG fragments, a partial complex formation of silica particles and the DNA might occur, although these interactions are not reported at neutral pH yet.

In contrast, the log-reduction was not observed in the absence of silica particles, which also supports the possibility of a complex formation. However, it should not be ruled out that the presence of the silica particles worsens the efficiency of the polymerase in qPCR, or complicates the attachment of SYBR-Green molecules for quantification, resulting in the artificial reduction of ARG/DNA fragments. As complementary information, phosphorus concentrations were determined by ICP-MS in all filtrates. However, the detected levels were close to the limit of detection and showed no trends between the various filtrates (Supplementary Table S1). The lacking correlation with the PCR results is likely due to the low DNA concentration and the lower selectivity of DNA detection *via* the phosphorus signal suffering from the variation of elemental blank levels and spectral interferences. In addition, PCR may be affected by matrix effects, but has the advantage of higher detection selectivity and higher sensitivity for ARGs.

**FIGURE 3:**
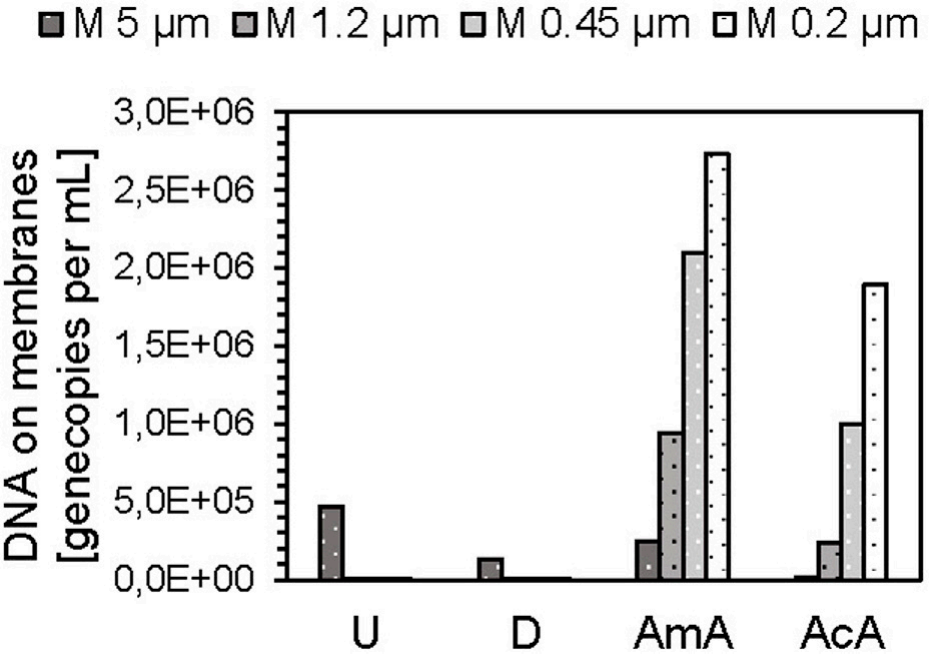
eARG/DNA fragments on membranes after DNA extraction of cascade filtration experiments with silica particles in ultrapure water (U, pH 7), drinking water (D, pH 7), amino acid buffer (AmA, pH 5), and acetic acid buffer (AcA, pH 5).

For the solutions with reduced pH (AmA, AcA, pH 5) and drinking water, the analysis by qPCR revealed slightly higher gene copy concentrations in the filtrates, compared to the feed. The reasons for that remain unclear, but probably non-optimal qPCR conditions due to high ion concentrations and lower pH might have led to impaired function of the polymerase during the PCR reaction. Therefore, the results of the DNA extracted from the membranes were considered here. An increasing trend with advancing CF was evident (Figure 3). Concentrations of eDNA fragments increase from 10^4.2^ (AcA) and 10^5.4^ GC mL^−1^ (AmA) on the 5 µm membrane to 10^6.3^ (AcA) and 10^6.4^ GC mL^−1^ (AmA) on the 0.2 µm membrane.

However, it remains unclear, whether the changing pattern of eDNA concentrations on the filters can be related to DNA–silica complexes.

From the qPCR analyses of the DNA retained on the membranes, it appears that the formation of DNA–silica complexes under neutral pH conditions plays a minor role compared to the buffers at pH 5. This supports the hypothesis that the particles only interfered with detection by qPCR but did not lead to aggregation with DNA molecules and certainly not to the retention of them. Since the ARG/DNA concentrations on the filters are significantly increased under the pH-reduced conditions (AmA and AcA), and increase further with the reduction of the pore size, this indicates increased aggregation between the silica particles and DNA, confirming the statements in the literature (Vandeventer et al., 2013).

In summary, the application of favorable conditions for the formation of DNA–silica complexes (application of certain buffers as matrix) and subsequent analysis by qPCR and ICP-MS turns out to be a major challenge. Reliable differentiation between DNA–silica and DNA–membrane interactions was not possible in these experiments. Similar to published studies, this methodology allows only for indirect characterization of potential DNA–particle interaction. Therefore, the feasibility of AF4 online with ICP-MS detection was explored in this study as an alternative method for size separation.

### AF4 Online With ICP-MS: Method Development for DNA Particle Adducts

#### Basic FFF Run of DNA Adduct Model System

The combination of silica particles and DNA fragments was also used as an initial sample for FFF-ICP-MS method development. However, naturally occurring clay minerals in a real surface-water matrix are much more suitable for future application, including natural ionic strength and organic compounds, which lacks the simple silica particle model. The observed results with the silica DNA samples are matching well with the results discussed in the following for surface water.

Stable model samples for method development were prepared by spiking calf-thymus (CT) DNA into environmental surface-water samples containing clay minerals. These suspensions were repeatedly prepared and kept stable for about 2 days allowing comparison of AF4 runs under different operating conditions with DNA-P levels well detectable by ICP-MS without the need for time-consuming fraction collection and offline PCR detection.

Neither the literature nor our initial cascade filtration experiment clearly indicated a suitable carrier composition for AF4. The typically applied buffer concentrations were too high for direct introduction into ICP-MS (Lee et al., 2001; Ashby et al., 2014; Griffith et al., 2020; Chowdhury et al., 2021) and significant post-channel dilution was not an option due to low ARG concentration in real water samples. However, it needs to be considered that environmental surface water is typically carbonate buffered with pH around 5 to 6 and contains various cations and low-molecular mass-organic acids that are likely to support DNA interaction with particles. In case of short duration of particle separation, low or even zero buffer concentration in the carrier might be sufficient. An experiment with 5 mmol L^−1^ calcium chloride in the carrier caused rapid decrease of the ICP-MS sensitivity combined with strong interaction of the silica particles with the PES membrane and was thus not further considered. Following our previous work on particulate phosphorus characterization in environmental water samples, 25 μmol L^−1^ NaCl was used as low-ionic-strength carrier with a 1 kDa PES membrane (Yang et al., 2020). The general verification of the applied ICP-MS method online with AF4 has been reported in previous work, including aspects of recovery and mass balance as well as the upper size limit for direct introduction of particles (Nischwitz and Goenaga-Infante, 2012; Heroult et al., 2014; Meermann and Nischwitz, 2018; Tan et al., 2020; Yang et al., 2020). The application to DNA particle adducts is novel and reported in this study for the first time. Reference samples of known composition are not available. The basic verification of the developed method is presented in the following sections, including systematic optimization and determination of the recoveries.

**FIGURE 4:**
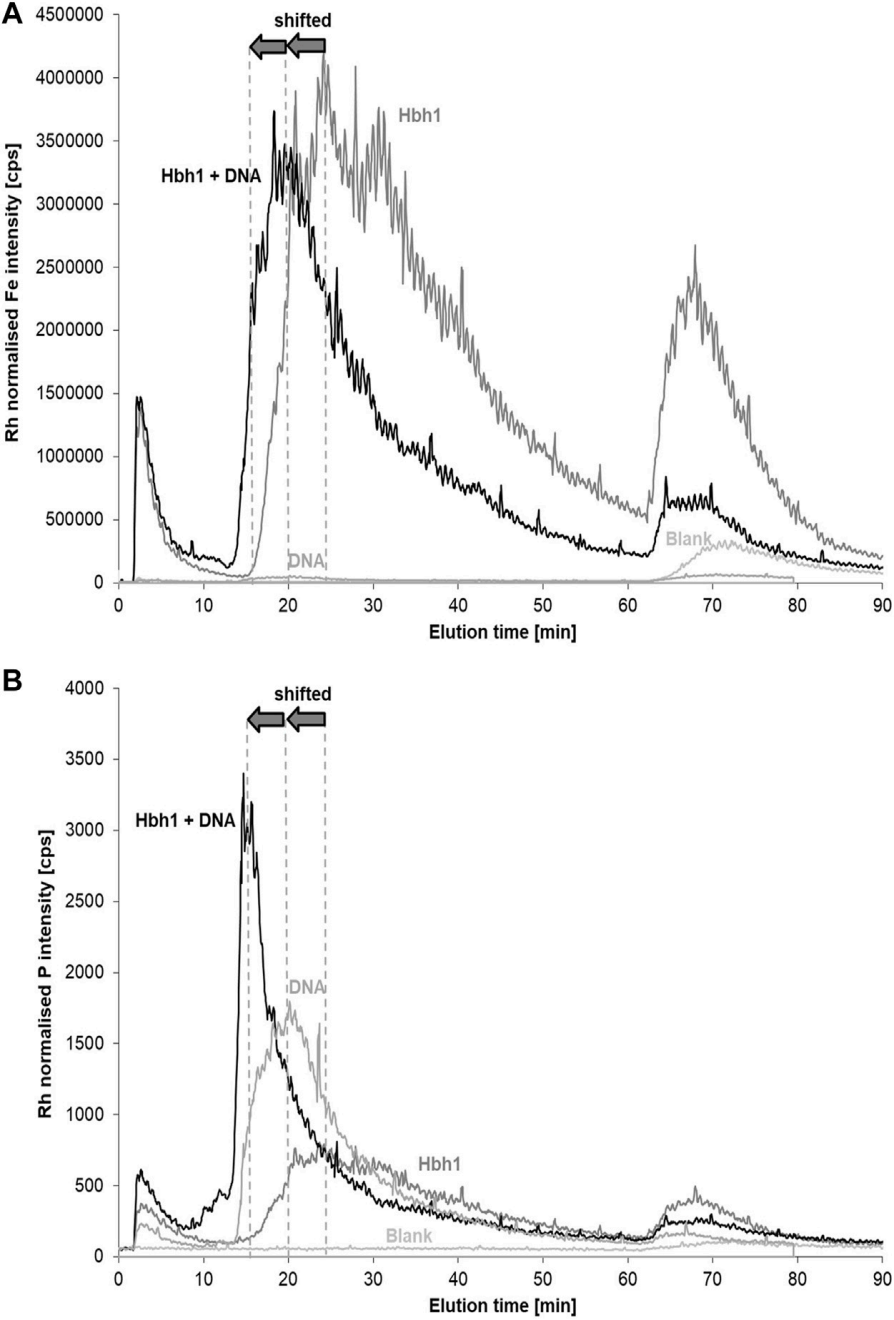
Fractograms of a surface-water sample (Hbh1), calf-thymus DNA in deionized water, the surface-water sample spiked with CT DNA, and a deionized water blank monitoring Fe **(A)** and P **(B)**. The carrier was 25 μmol L^−1^ NaCl and the membrane 1 kDa PES.

The cross flow was reduced from 2 mL min^−1^ to 1 mL min^−1^ to improve recovery without significant loss of peak resolution. These AF4 conditions showed clear differences between the original water sample and the same sample spiked with CT-DNA. The clay fraction as monitored by Fe (and in analogy by Al and Si) partially shifted to earlier elution times and the spiked DNA appeared in the phosphorus signal as increased peak overlapping with the fronting of the shifted clay fraction (Figure 4). Obviously, the DNA changed the clay surface by partial binding. However, the formed adduct appeared at lower instead of higher elution time as expected for larger particle size. This leads to the conclusion that the formed aggregate is partially rejected from the membrane of the channel due to charge effects or due to the steric effect of the DNA molecule(s) attached to the surface. When overlaying the P and Fe signals for the original sample, there is a perfect match of the peak shapes (Supplementary Figure S3A). In case of the spiked sample, the P fraction has much smaller peak width and overlaps only with the fronting of the broader Fe fraction (Supplementary Figure S3B), indicating that the clay particles are only partly modified by the DNA at the spike level applied in this experiment.

Separate injection of CT-DNA at the same concentration without environmental water matrix resulted in a phosphorus peak eluting a bit later and broader than the emerging peak in the spiked sample. The higher ionic strength in the environmental sample is likely to support the elution of the DNA. A potential artifact may occur due to DNA partially covering the surface of the membrane and thus affect clay particle elution without being bound to the clay particles. However, the DNA P signal of the free DNA and the spiked DNA in the water matrix elutes without unusual tailing, and there was no gradual increase of the phosphorus baseline observed as an indicator of delayed elution of membrane-attached DNA. The relative peak area of the final fraction after the release of the cross flow to zero is even smaller in the spiked sample and free DNA compared to the original sample.

Further evidence is provided by repeated analysis of the original non-spiked water sample after the spiked sample. The elution profile of Fe is exactly matching with the first fractogram of the sample (Supplementary Figure S4). This demonstrates that the shifted elution time for the DNA-spiked water sample is due to direct DNA–clay interaction and not an artifact of DNA-coated membrane on clay elution.

#### Variation of Carrier Composition and Membrane

The initial results suggest partial binding of DNA to clay particles. However, the unusual elution order and potential instability of DNA–clay adducts in the non-buffered low-ionic-strength carrier or due to interaction with the membrane require more detailed investigation to exclude artifacts. Therefore, two other membrane materials, 1 kDa regenerated cellulose (RC) and 50 kDa polyvinylidene fluoride (PVDF), were applied with the same carrier and same model samples.

**FIGURE 5:**
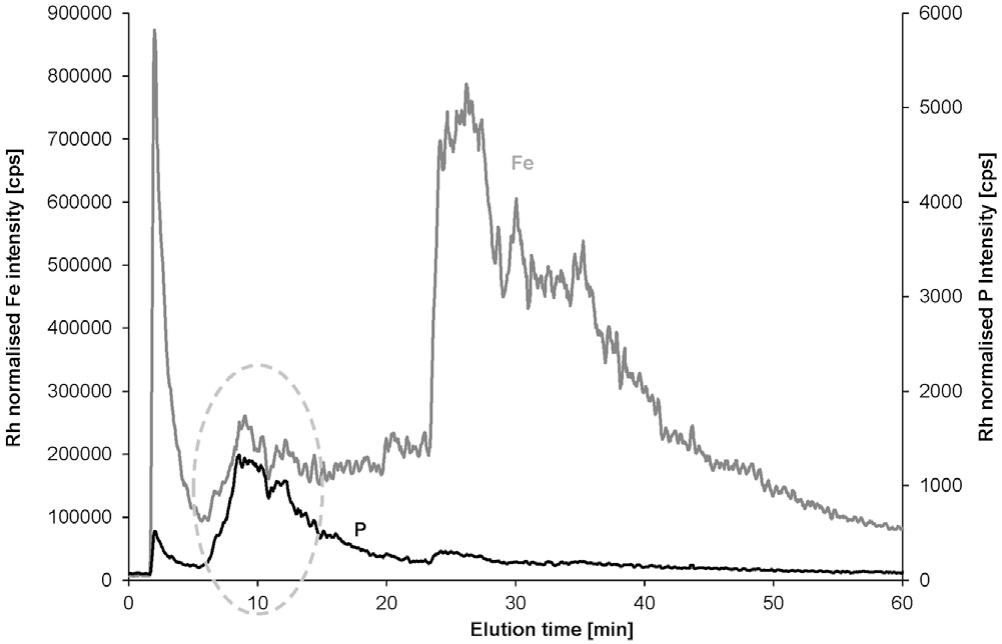
AF4 separation of the DNA-spiked water sample Hbh1 using 50 kDa PVDF membrane and 10 min longer constant cross flow before the gradient (all other parameters were the same as for the data in Figure 4).

The fractograms with the 1 kDa RC membrane showed very similar elution profiles and elution times for the spiked model sample compared to the 1 kDa PES membrane using the 25 μmol L^−1^ NaCl carrier (Supplementary Figure S5). PVDF was not available at 1 kDa pore size and thus applied in 50 kDa pore size with the same carrier and same cross flow for the DNA-spiked water sample (Supplementary Figure S5). In contrast to the PES and RC membranes, the elution profile significantly changed and a separate fraction was detected between the void peak and the original clay peak both for Fe and P. The expected lower interaction with the non-functionalized PVDF membrane enabled much better separation of the DNA containing fraction—as indicated by the phosphorus signal—from the remaining original clay fraction represented by the Fe signal. Also, the new fraction is represented with a matching peak profile in the Fe fractogram, most likely indicating the presence of DNA–clay adducts. The method was further developed by keeping the cross flow constant for 10 additional minutes before the gradient. This improved the separation of the new fraction from the original clay fraction (Figure 5).

Apart from the membrane, the carrier composition was varied by using 5 mmol L^−1^ ammonium acetate (pH 7) with the 1 kDa RC membrane. This results in similar peak pattern as observed with the NaCl carrier, but with larger peak width and much later elution times. The same pattern was obtained when using 5 mmol L^−1^ Tris buffer (pH 5) with the 50 kDa PVDF membrane (Supplementary Figure S6). When using buffered carriers, the interaction of the spiked DNA with clay particles also causes a shift of Fe to a new earlier eluting fraction with a matching P signal. However, due to the much stronger interaction with the membrane, the recoveries are much lower compared to the NaCl carrier.

Percentage recoveries of P and Fe in the size-separated samples were calculated using flow injection of the same samples to determine to the total injected elemental concentrations (Table 2).

**TABLE 2:** Recoveries [%] for AF4 separation of non-spiked and spiked water samples (*n* = 2; DNA in water *n* = 1).

Membrane/Carrier	Element	Non-spiked Sample (Hbh1)	DNA Spiked Sample (Hbh1)	DNA in Water
1 kDa PES/25 μmol L^−1^ NaCl	P	72 ± 4	55 ± 1	97
	Fe	93 ± 4	52 ± 9	-
1 kDa RC/25 μmol L^−1^ NaCl	P	55 ± 9	67 ± 5	106
	Fe	61 ± 18	54 ± 12	-
50 kDa PVDF/25 μmol L^−1^ NaCl	P	78 ± 3	79 ± 5	110
	Fe	111 ± 8	69 ± 6	-
50 kDa PVDF/5 mmol L^−1^ Tris	P	15 ± 1	59 ± 2	103
	Fe	20 ± 2	33 ± 1	-

Considering the faster elution, smaller peak width, better recoveries, and, in principle, the same elution pattern, the original carrier 25 μmol L^−1^ NaCl with the 50 kDa PVDF membrane was chosen as optimum conditions for subsequent experiments.

#### Variation of the DNA Spike Level

**FIGURE 6:**
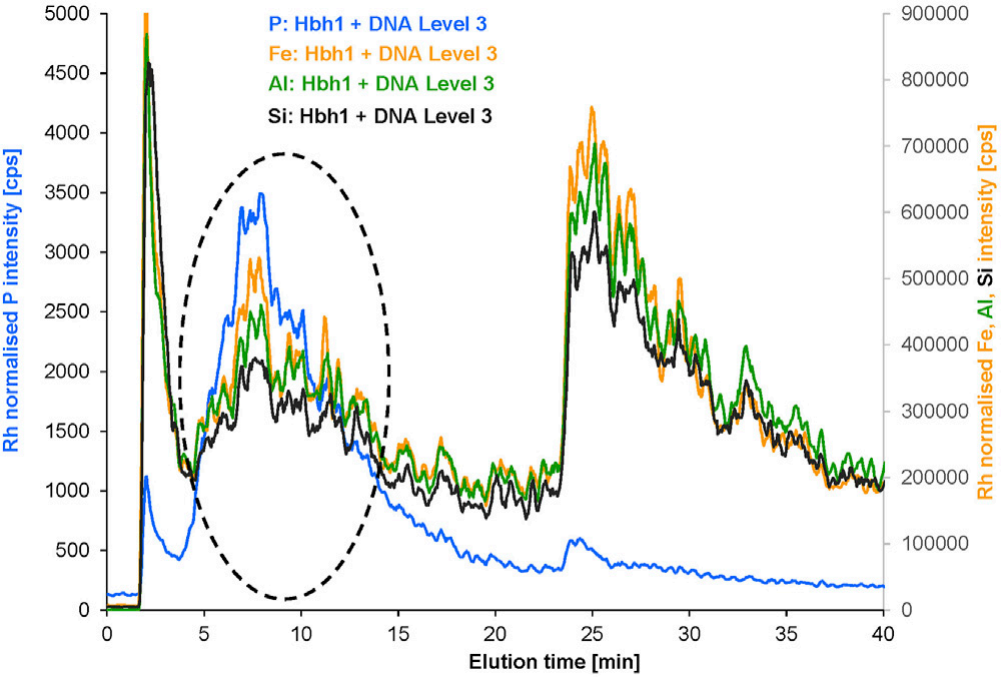
Overlayed fractograms of P, Fe, Al, and Si for the Hbh1 water sample at DNA spike level 3 (the signals of Al and Si were multiplied by scaling factor 9 to match the Fe intensity range).

The initial spike level was selected to ensure sufficient DNA concentration to allow detection of potentially formed DNA clay adducts *via* the phosphorus signal by ICP-MS. The partial shift of the Fe signal representing clay particles to the new fraction was observed. To study the DNA interaction with the clay in more detail, the spike level was increased by a factor of 2 and 3, respectively (Supplementary Figure S7). The results show increasing Fe and P concentrations in the new shifted fraction suggesting a high capacity of clay for DNA binding. Most importantly, the peak profiles of Fe and P in the new fraction match very well, supporting that both elements are bound within the same particulate species and are not accidentally coeluting (Figure 6). Apart from the new fraction marked by a dashed circle, there is also a minor P peak observed in the void fraction at a much lower P-to-Fe ratio. Interpretation of the void peak in AF4 is critical because steric elution of large particle aggregates may overlap with normal elution of small particles. Therefore, the void peak was not further evaluated at this stage.

Further evidence for the presence of clay in the new fraction is provided by the Al and Si profiles exactly matching with Fe both in the remaining original fraction and in the new fraction. This is clear proof of the intact particles shifting to the new fraction without dissociation. The reason for this shift and co-elution with P is directly related to the DNA spike level due to the surface interaction of DNA with clay.

The quantitative evaluation of the elemental composition of the original fraction and the new fraction (Supplementary Figure S7) for the increasing DNA spike level of the water sample Hbh1 supports this conclusion. The phosphorus mass in the new fraction is increasing by 268% proportional to the DNA spike level 1 to 3, which is consistent with the peak assignment to DNA and DNA–clay adducts (Supplementary Figure S8). The Al/Fe ratio remains constant over this range of spike levels both for the original fraction and for the new fraction with a change of only 2 and 6%, respectively. This clearly indicates that the new fraction contains inorganic colloids of the same composition as the original fraction, which were shifted to earlier elution time due to surface modification by interaction with DNA. Moreover, the P/Al ratio in the new fraction is nearly constant with an increase of only 26% from spike level 1 to 3. This indicates a defined ratio of DNA to clay in the formed adducts, but it needs to be kept in mind that the free DNA is coeluting and thus the ratio of bound DNA to clay cannot yet be calculated from these data.

The molar Si/Al ratio for the original fraction is in the range of 2.14 to 2.21 and for the new fraction in the range of 2.00 to 2.07, which agrees well with a 2:1 layered clay mineral (Chatterjee et al., 1998; Bourg et al., 2007).

#### Spike Experiments With eARG Fragments

**FIGURE 7:**
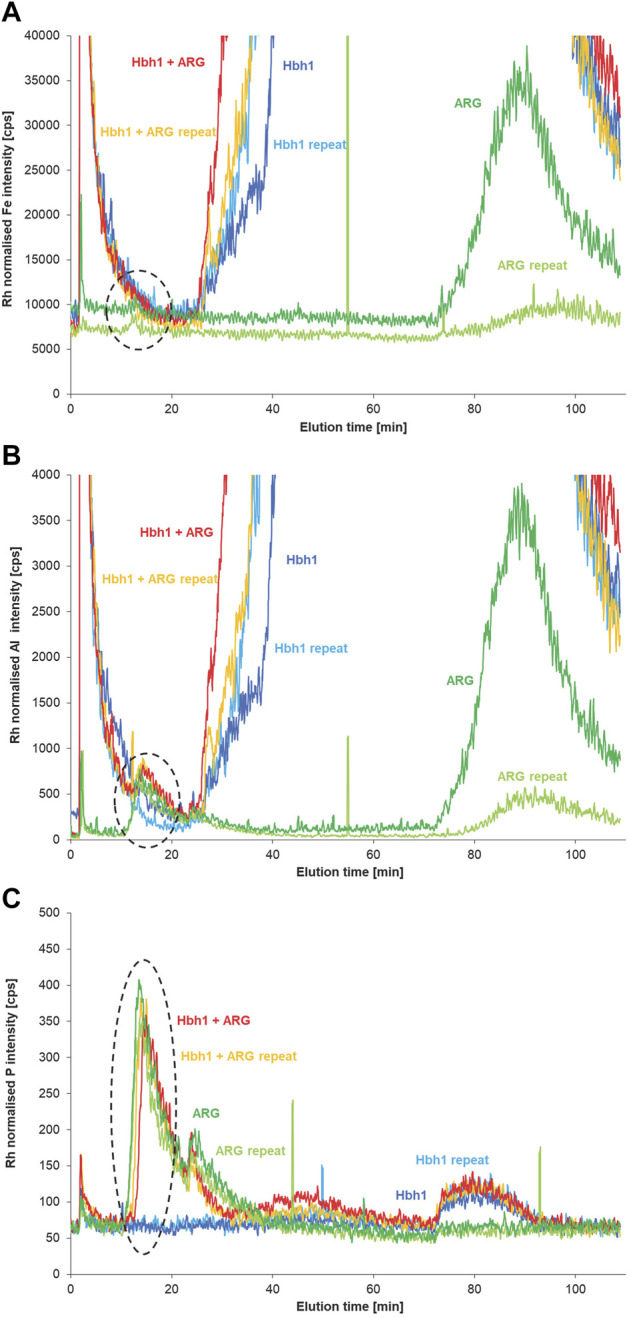
AF4 fractograms of the natural water sample (Hbh1), the sample spiked with eARG fragment of 530 kDa, and the fragment in deionized water monitoring Fe **(A)**, Al **(B)**, and P **(C)** by ICP-MS (*n* = 2).

Following optimization of the most relevant parameters of the developed FFF separation method by using the easily available calf-thymus DNA, the method was applied to water samples spiked with eARG fragments. Due to limited amount of the eARG, only a few experiments were possible at this stage. Analysis of the two fragments of 56 and 530 kDa without clay matrix demonstrated that the AF4 method achieves separation of the DNA according to molecular size (Supplementary Figure S9). The smaller eARG fragment of 56 kDa elutes rather early, and thus in the spiked water sample, the potentially formed clay DNA fraction is partly overlapping with the void peak and not clearly detected at the low spike level. In case of the larger fragment of 530 kDa, the DNA fraction is sufficiently separated from the void peak (Figure 7). The phosphorus signal shows clear peaks of the eARG with and without the water matrix at about 14 min. Matching signals at this elution time are also observed for the aluminum signal with clear increase for the ARG-spiked sample compared to the non-spiked sample, suggesting an interaction of ARG with clay particles in analogy as observed for the CT-DNA spike experiments. In case of iron, the baseline intensity is rather high and there is no difference between the spiked and non-spiked sample visible.

However, for the ARG fragment in deionized water, there is also an aluminum peak observed at a retention time of 14 min matching the P signal. This might be due to the fact that the ARG fragment is binding already ionic aluminum prior to the FFF separation. Another more likely explanation is a binding of the ARG fragment to residual clay particles present on the membrane in the FFF channel from the previous run of the non-spiked water sample. This is supported by Fe and Al peaks at zero cross flow (about 90 min elution time) that are observed for the ARG and at decreasing levels for the subsequent repeated analysis of ARG while there is no P peak eluting at this time. This indicates residual clay, which is critical at these low ARG levels but not for the previous experiments with CT DNA at higher spike levels. To exclude artifacts from low-molecular-mass Al-binding to free ARG, it is required to separate the free ARG from the particulate ARG, as further discussed in the following section.

#### Separation of DNA From DNA–Clay Adducts

A slight disadvantage of the current method is the fact that the free DNA as analyzed by fractionation of an aqueous DNA solution at the same concentration level as spiked into the Hbh1 water sample elutes nearly at the same time as the new DNA clay fraction in the spiked water samples (Figure 4 and Figure 7). Therefore, it is not possible to distinguish the free DNA from the particle-bound DNA.

To overcome this limitation, the pore size of the PVDF membrane was increased to 150 kDa to enable the free DNA to pass through the membrane during focusing and be removed from the separation channel *via* the cross flow. This approach was investigated with a small eARG fragment of 56 kDa molecular weight. However, the phosphorus peak of the free eARG fragment in water was at the same level and elution time as for the Hbh1 water sample spiked with this fragment, indicating that the eARG fragment is not passing through the membrane. This could be due to the fact that the molecular weight cut off of the membrane typically refers to the spherical shape of the molecule, while eARG is likely to be present as a long partially folded polymer chain. In addition, electrostatic interaction may contribute to the observed effect.

**FIGURE 8:**
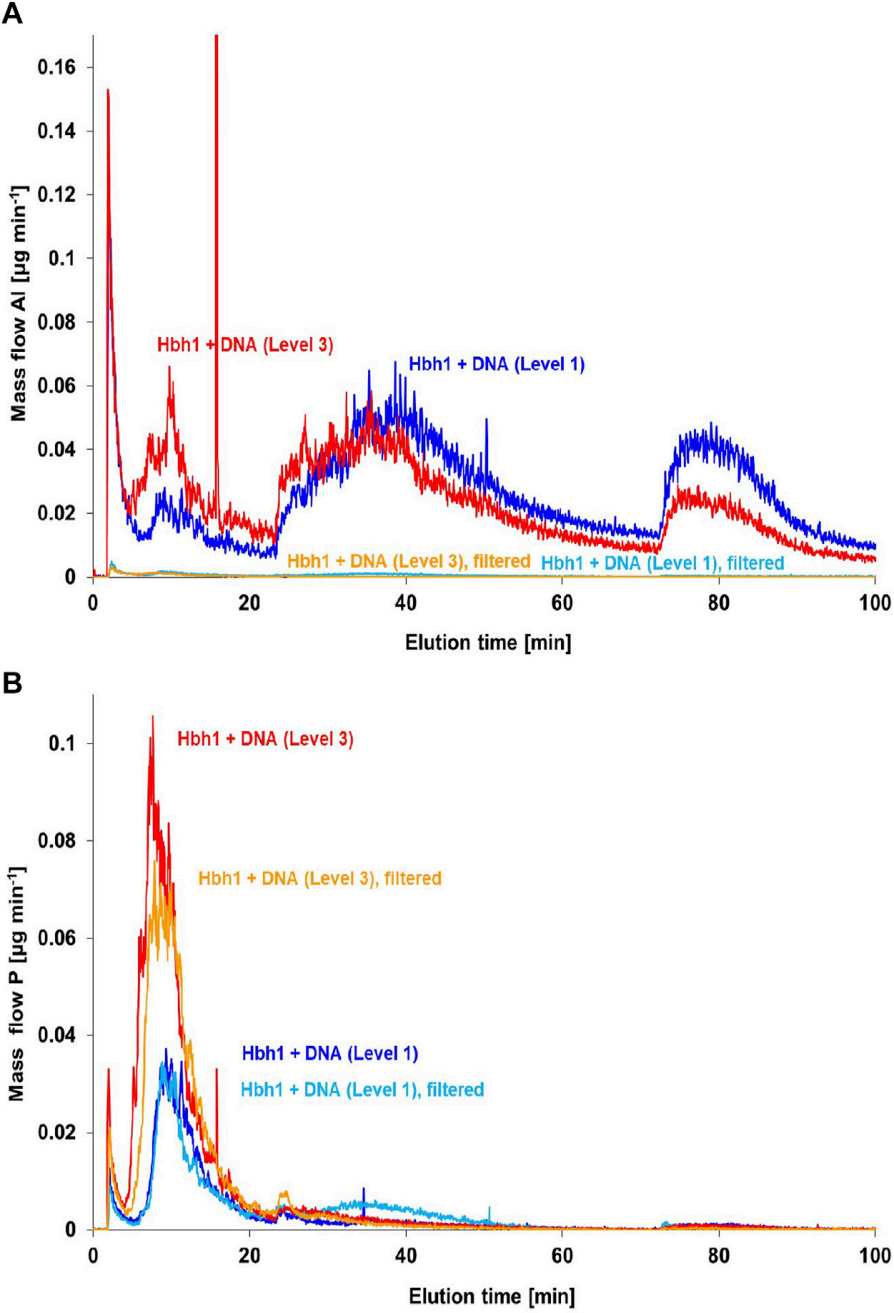
Post-channel filtration for the separation of DNA–clay adducts from free DNA in CT-DNA-spiked water samples at two concentration levels (1) and (3) monitoring Al **(A)** and P **(B)**.

Alternatively, a 100 nm inline membrane filter was inserted between the FFF channel and the ICP-MS to retain the clay and clay–DNA adducts from entering the ICP-MS and thus monitoring only the phosphorus signal of the free DNA. Initially, this experiment was performed with the basic calf-thymus DNA spike level 1 (Figure 8). The aluminum fractograms clearly indicate that the clay minerals are retained by the post-channel filter, resulting in practically blank signal matching the DNA in deionized water. However, the phosphorus fractograms show hardly any change, suggesting that the amount of DNA bound to clay is rather low. Quantitative evaluation reveals a difference of about 3%, which is within the uncertainty of the quantification. Therefore, the experiment was repeated with higher DNA spike level 3. In this case, the phosphorus signal in the relevant fraction around 14 min elution time is clearly decreased when using the post-channel filtration. The change accounts for about 15% of phosphorus in this fraction, which is in the same order as estimated in previous incubation studies (Pietramellara et al., 2007).

The key message of this experiment, however, is the fact that the matching aluminum peak at the DNA elution time is completely retained by the 100 nm filter, proving that the aluminum is bound within clay particles and not just complexed in ionic form directly by the DNA molecule. This observation strongly indicates formation of DNA–clay adducts in the investigated natural water sample spiked with CT-DNA.

## Conclusion

The complementary application of two particle-size fractionation techniques with PCR and/or ICP-MS detection provided new possibilities for direct characterization of DNA–particle interaction in model systems and real surface-water matrices. The cascade filtration has, in principle, the limitation that membrane clogging or membrane particle interaction may result in the retention of particles or compounds below the molecular size cut off. The advantage of field-flow fractionation is the particle separation in a thin channel without a stationary phase. The membrane at the bottom of the channel is only required to pass the low-molecular-mass species and the solvent. During the separation and elution, the particles are continuously purged through the channel above the membrane, and thus clogging of the membrane is not an issue. Therefore, particle membrane interaction may affect the elution time depending on particle surface but is less critical for size separation compared to cascade filtration.

FFF separation with multi-element detection by ICP-MS provided reliable characterization of the clay–DNA adducts. Using the novel approach of post-channel filtration also separation of free calf-thymus DNA and clay–DNA adducts could be achieved. The results of this initial method development and feasibility study will be further investigated in future applications.

## Data Availability

The original contributions presented in the study are included in the article/Supplementary Material, further inquiries can be directed to the corresponding author.
