# Supplementary material for: Cascade Filtration With PCR Detection and Field-Flow-Fractionation Online With ICP-MS for the Characterization of DNA Interaction With Suspended Particulate Matter
Source: Front Chem. 2022 Jun 28;10:919442. doi: 10.3389/fchem.2022.919442 (PMC9274009; doi:10.3389/fchem.2022.919442)

Supplemental material

Table S1 Si and P quantified by ICP-MS in the filtrates of the experiments with silica particles and eARG-fragments in ultrapure water.

filtrate	**Si [mg/L]**	**P [mg/L]**
< 5 µm	750 (± 30)	0.023 (± 0.005)
< 1.2 µm	750 (± 30)	0.021 (± 0.005)
< 0.45 µm	152 (± 7)	0.02 (± 0.01)
< 0.2 µm	46 (± 3)	0.022 (± 0.007)

Table S2 Physico-chemical parameters for the surface water samples from river Alz

	May 2021	August 2021
Conductivity [µS cm^{-1}]	n.d.	405
Water Temperature [°C]	13.4	18.6
pH	n.d.	8.7
O_2 concentration [mg L^{-1}]	n.d.	6.0

n.d. not determined

Figure S1 Sum of genecopy concentrations (GC/mL) on all filters (5 µm, 1.2 µm, 0.45 µm and 0.2 µm, black columns) compared to genecopy concentrations obtained by the standard filtration (blue/white plaid columns) method using only a 0.2 µm PES membrane for both surface water samples E1 (May 2021) and E2 (August 2021). Only detectable ARGs are shown. Other investigated ARGs like *vanA*, *blaCTX-M-32, blaSHV*, *mecA* and *mcr-1* were below the LOQ (1 genecopy per mL).

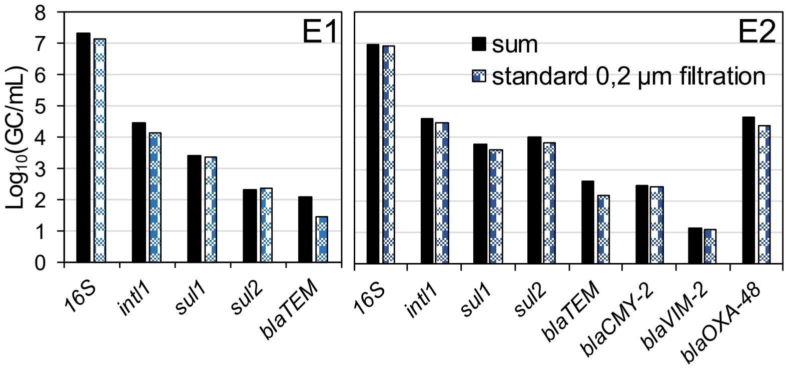

Figure S2 Relative quantification of commercially available silica nanoparticles of 100 nm, 500 nm, 1000 nm and 3000 nm particle size via flow injection ICP-MS (using the AF4 autosampler with a carrier of 25 µmol L^{-1} NaCl).

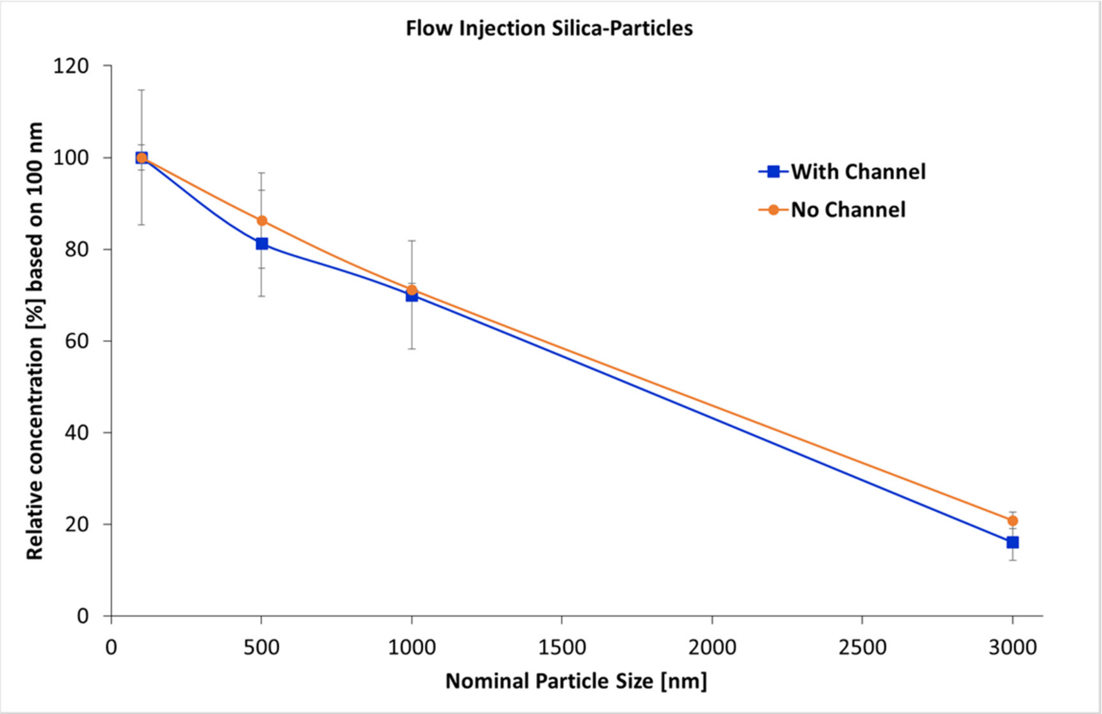

Figure S3 Overlay of the Fe and P signals for the original water sample Hbh1 (a) and the same water sample spiked with calf thymus DNA (b). AF4 separation was performed with 1 kDa PES membrane with 25 µmol L^{-1} NaCl carrier at cross flow of 1 mL min^{-1}.

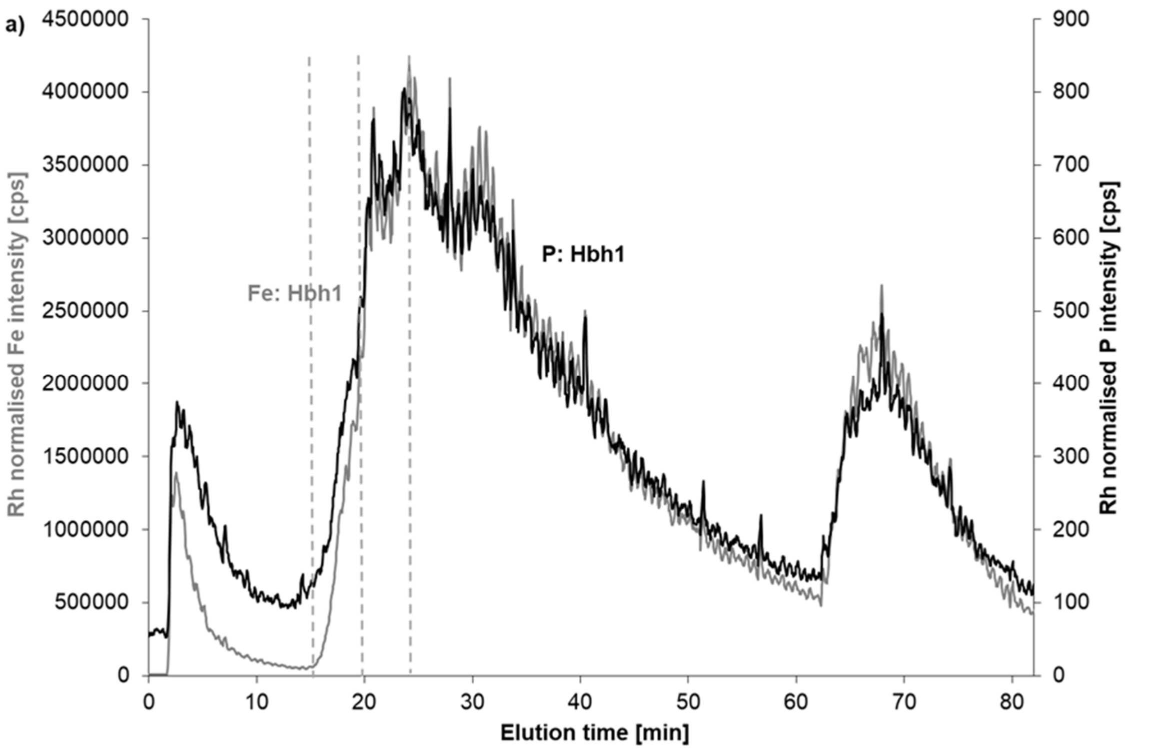

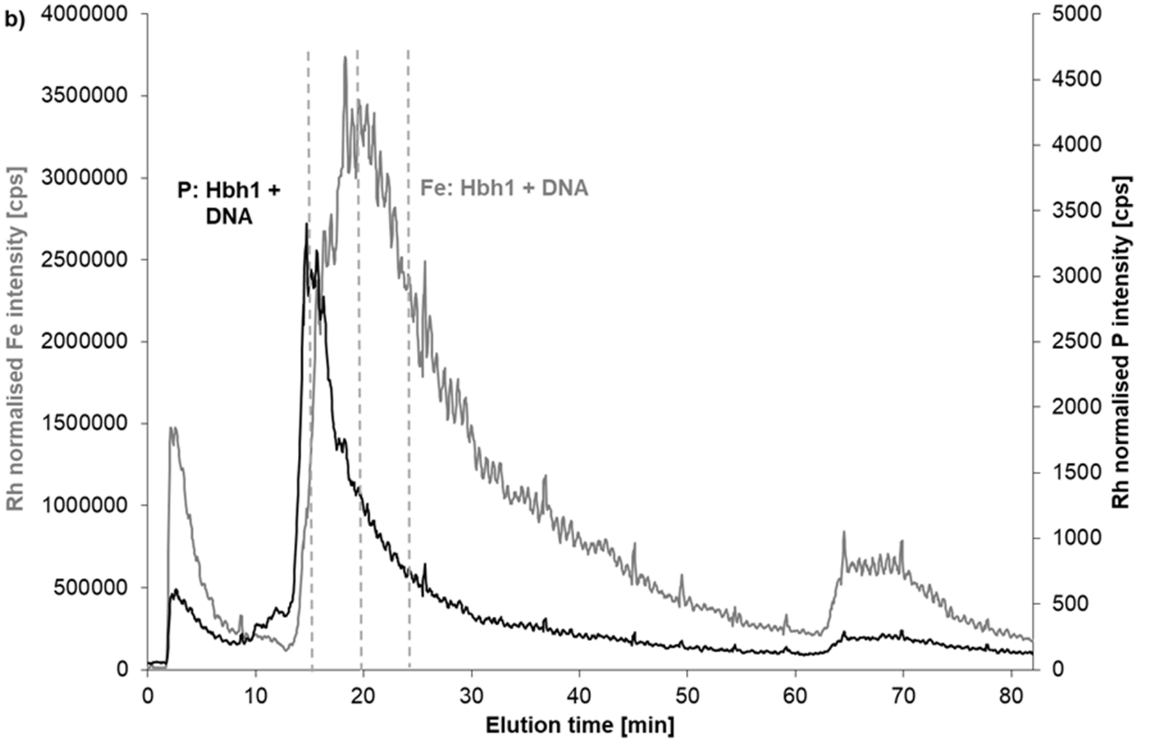

Figure S4 Repeated analysis of the non-spiked water sample (light blue) after the spiked water sample (red) demonstrating exact matching of elution times and peak profiles with the first analysis of the non-spiked water sample (dark blue) at the beginning of the sequence. Same AF4 conditions as in Figure 1 and Figure S1.

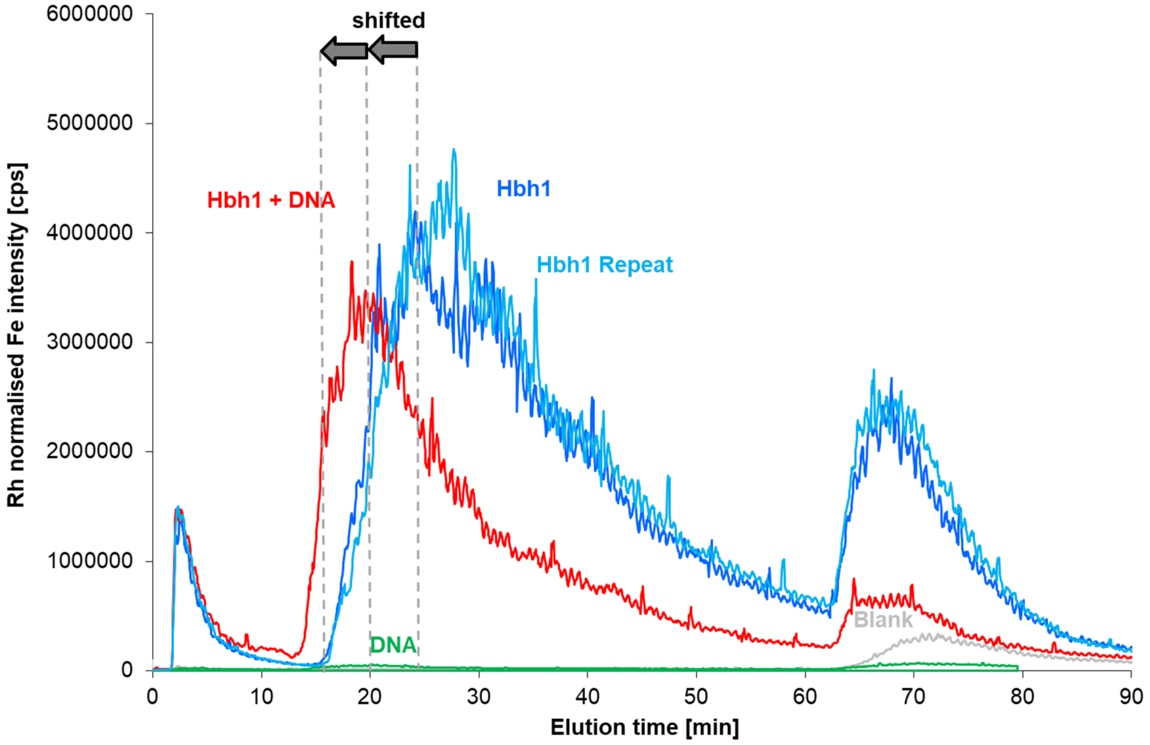

Figure S5 Comparison of 1 kDa PES membrane, 1 kDa RC membrane and 50 kDa PVDF membrane for the DNA spiked water sample using 25 µmol L^{-1} NaCl as carrier.

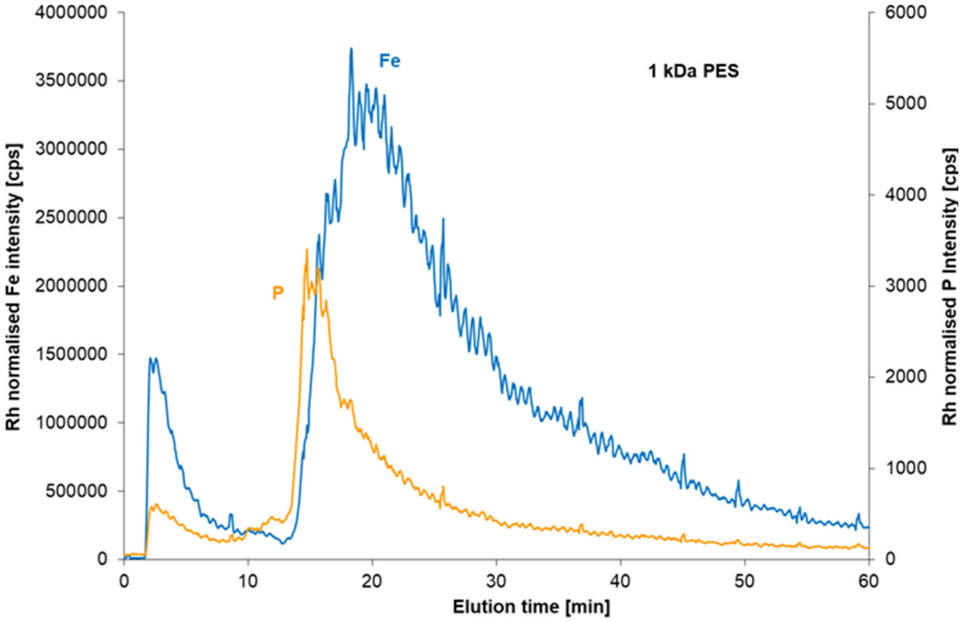

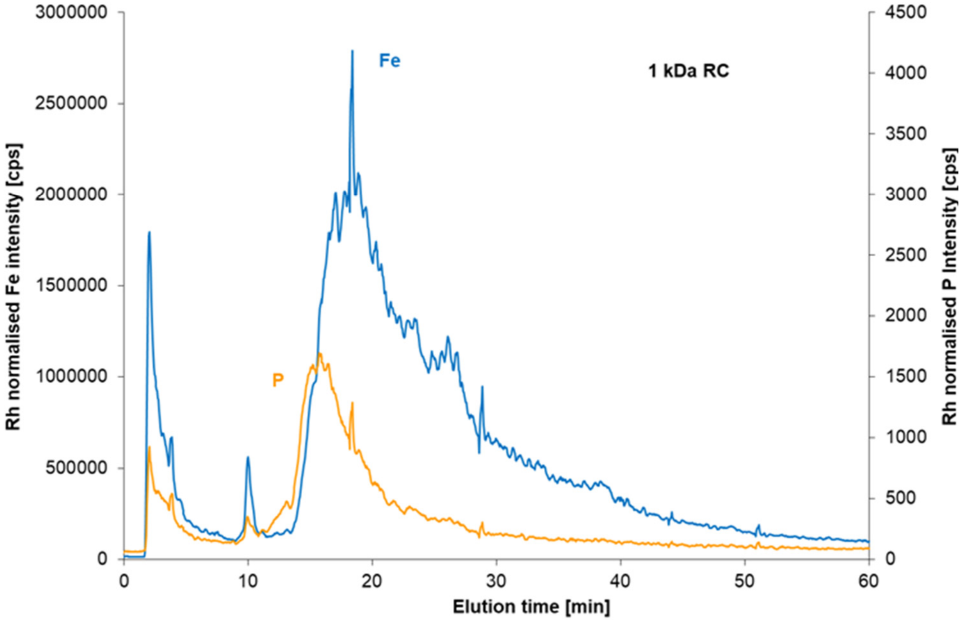

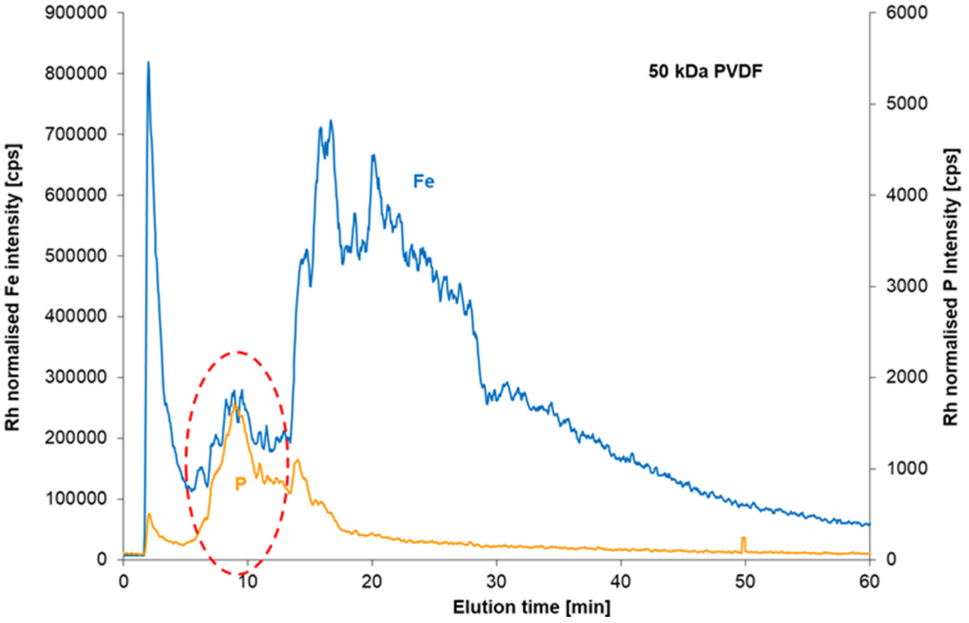

50 kDa PVDF
Fe
P
Rh normalised Fe intensity [cps]
Rh normalised P Intensity [cps]
Elution time [min]
900000
800000
700000
600000
500000
400000
300000
200000
100000
0
6000
5000
4000
3000
2000
1000
0
0
10
20
30
40
50
60

Figure S6 Fractograms of the non-spiked water sample (Hbh1), the DNA spiked water sample, DNA solution and blank for 50 kDa PVDF membrane with 5 mmol L^{-1} Tris buffer (pH 5) as carrier showing Fe (upper graph) and P (lower graph).

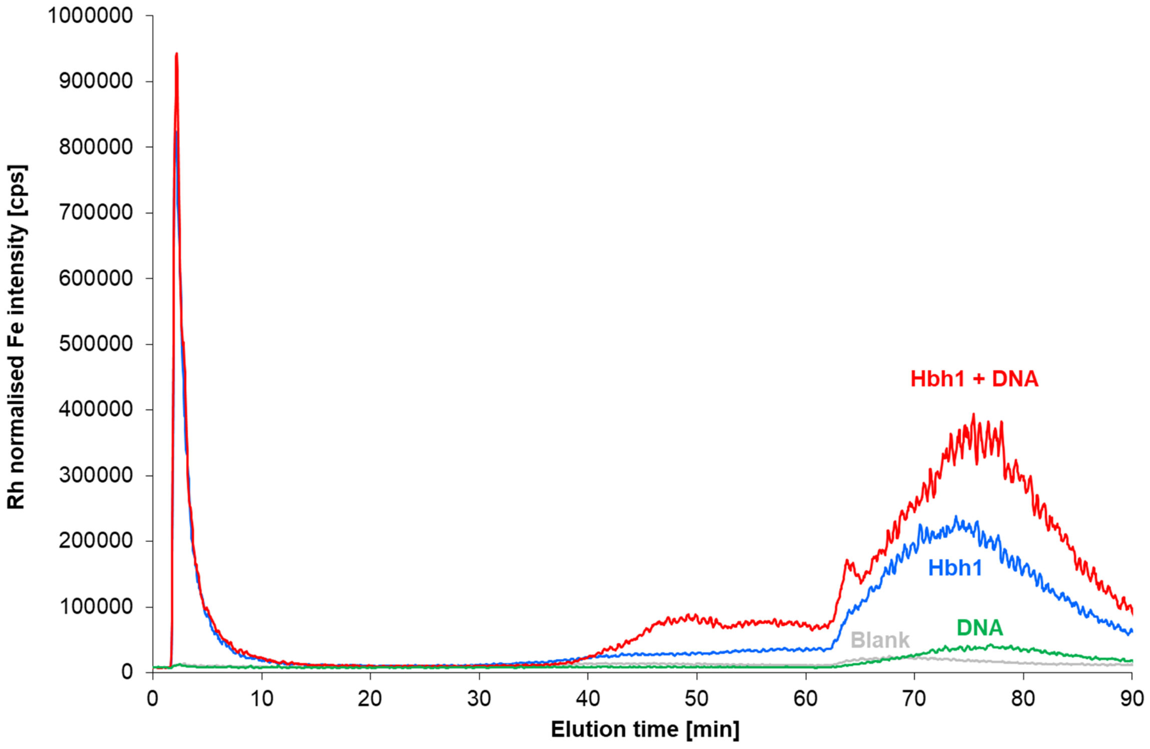

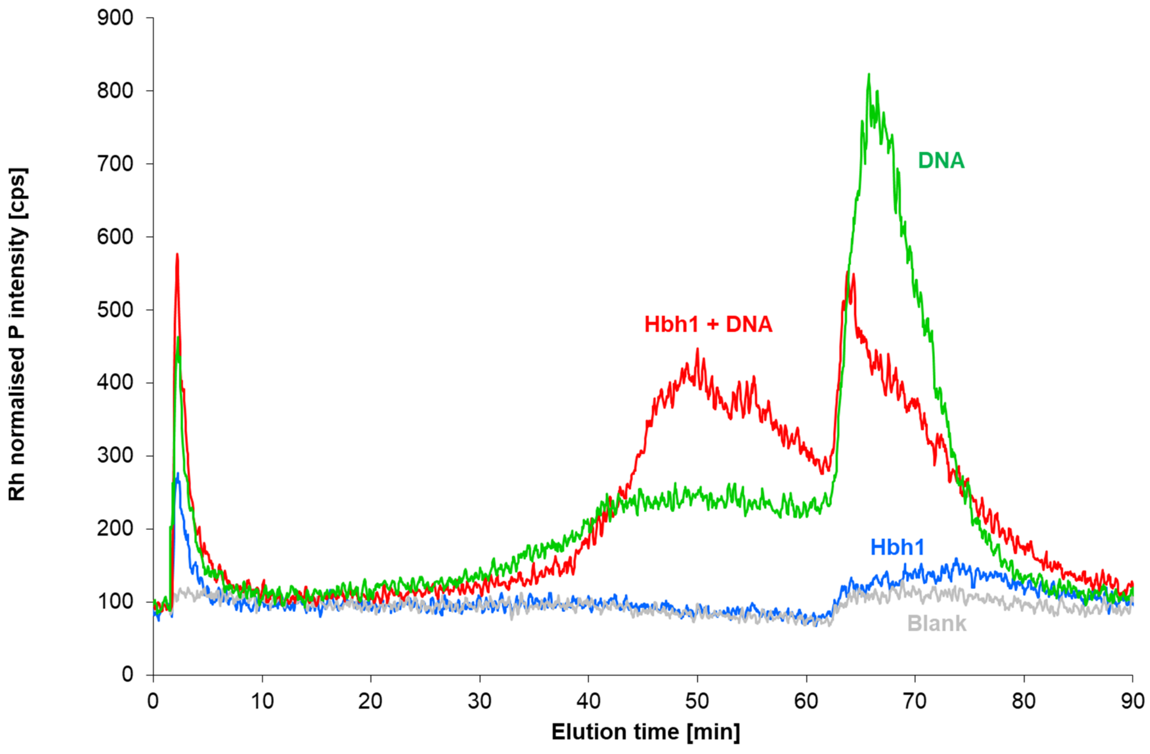

Figure S7 Variation of the DNA spike level for the water sample Hbh1 using 50 kDa PVDF membrane with 25 µmol L^{-1} NaCl carrier monitoring Fe (upper graph) and P (lower graph)

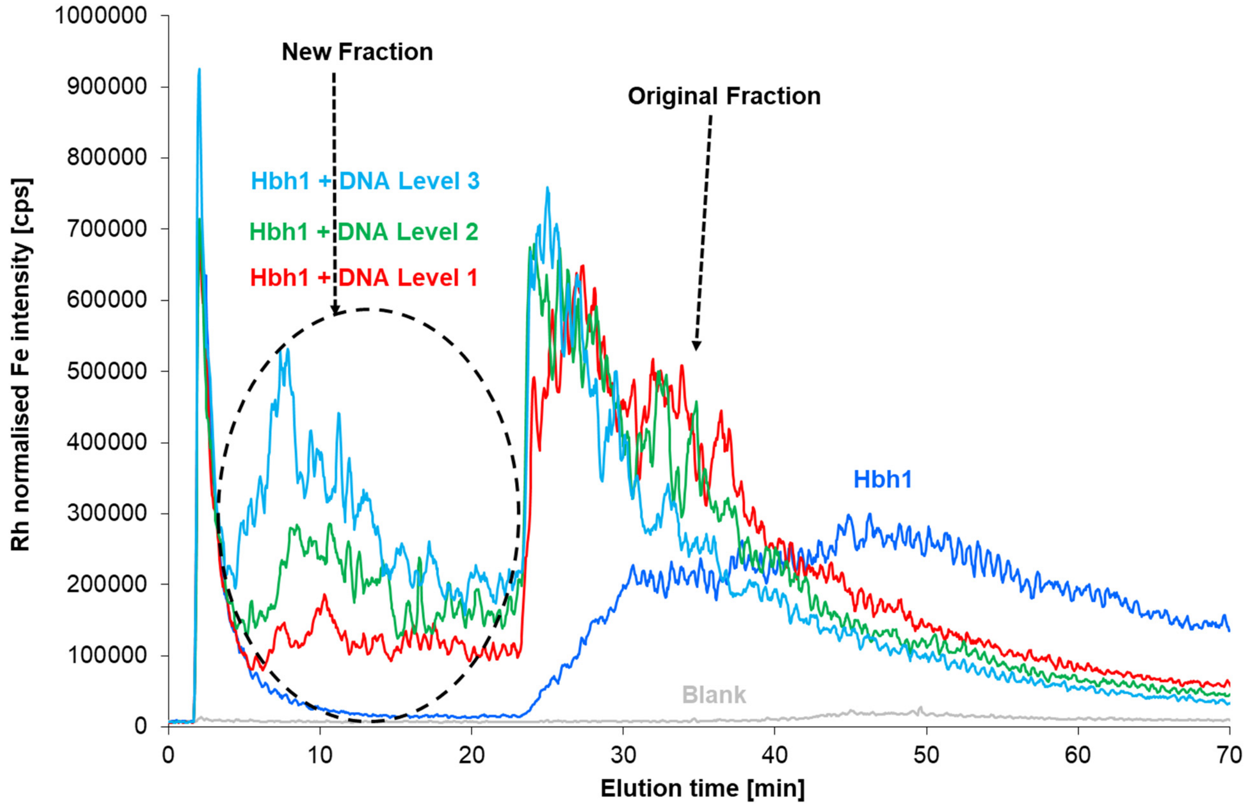

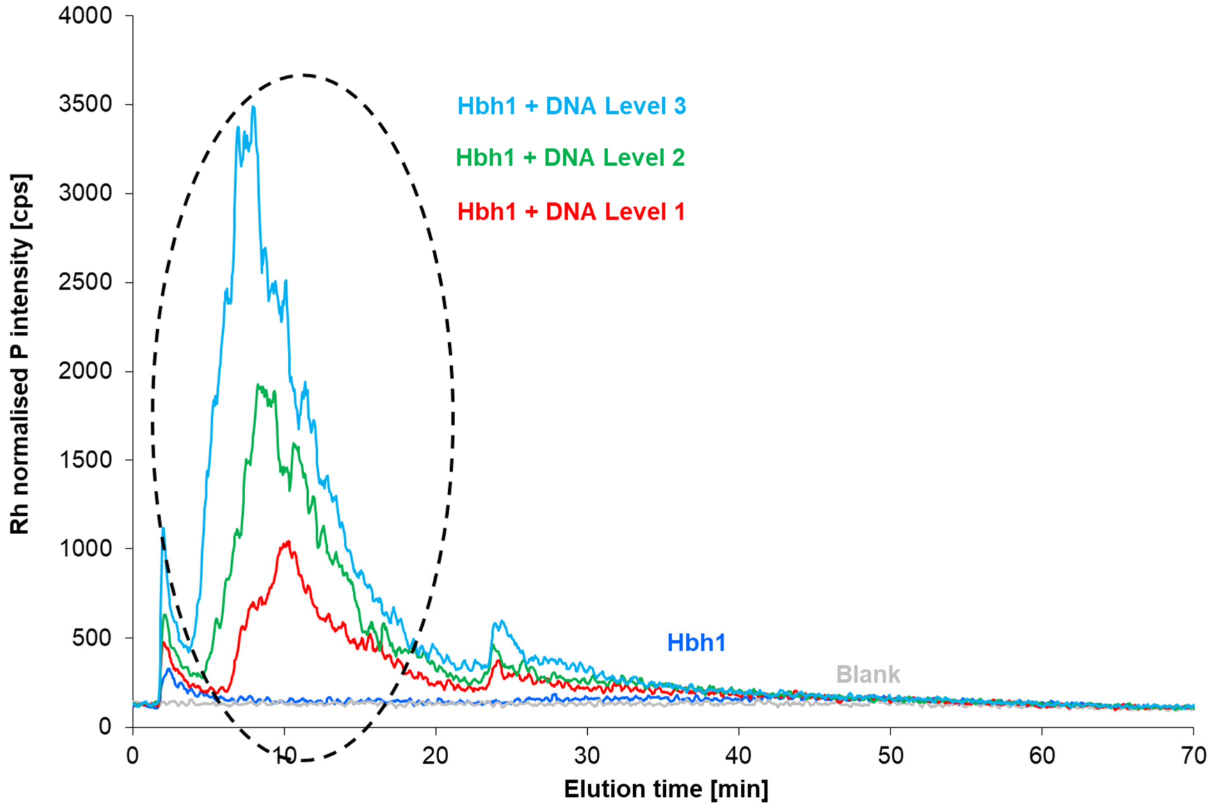

Figure S8 Quantitative evaluation of the phosphorus mass in the new fraction (dashed circle in Figure S7) as a function of the increasing DNA spike level for the water sample Hbh1. In addition, the P/Al ratio in the new fraction as well as the Al/Fe ratio in the new and in the original fraction are shown. At spike level 0 (original sample) there is no peak at the elution time of the new fraction and therefore the elemental ratios are not included here.

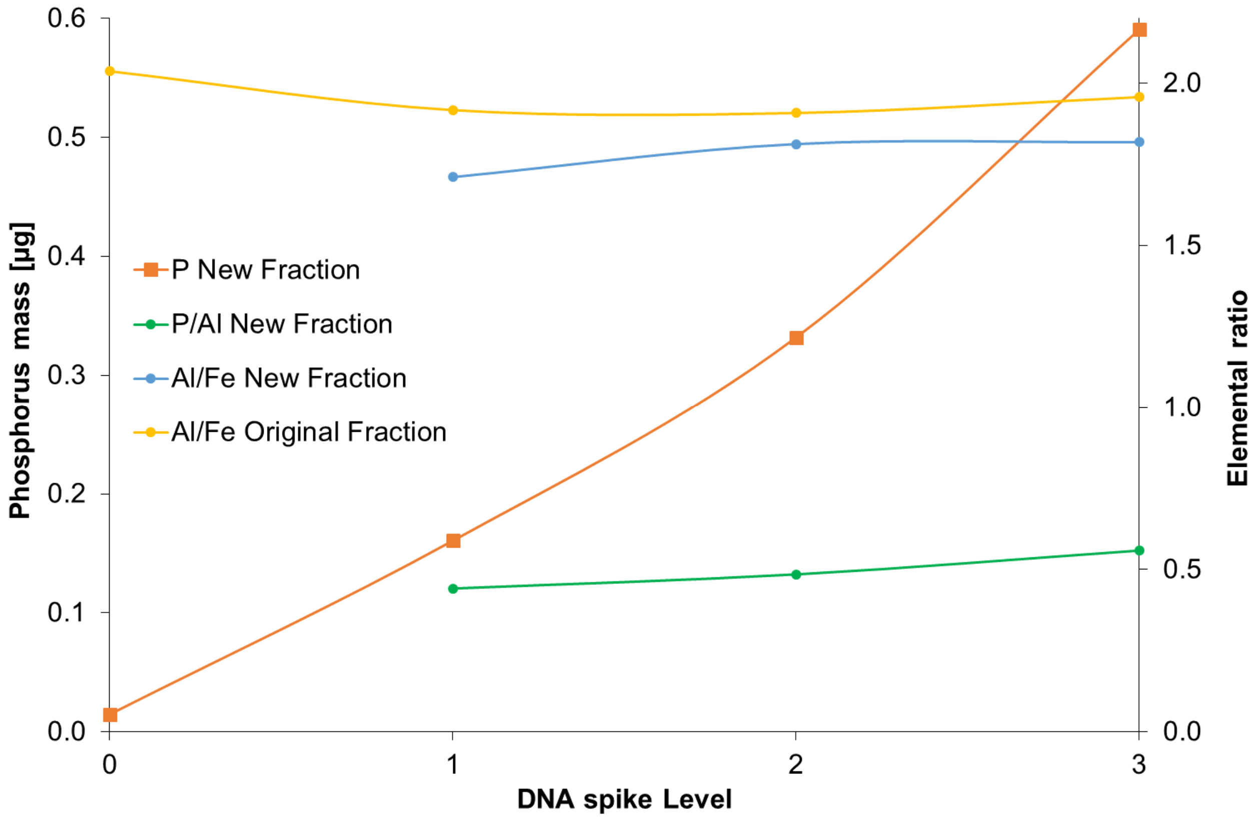

Figure S9 Size separation of DNA fragments 56 kDa, 530 kDa and calf Thymus DNA by AF4 online with ICP-MS for detection via the P signal (n=2 for the 530 kDa)

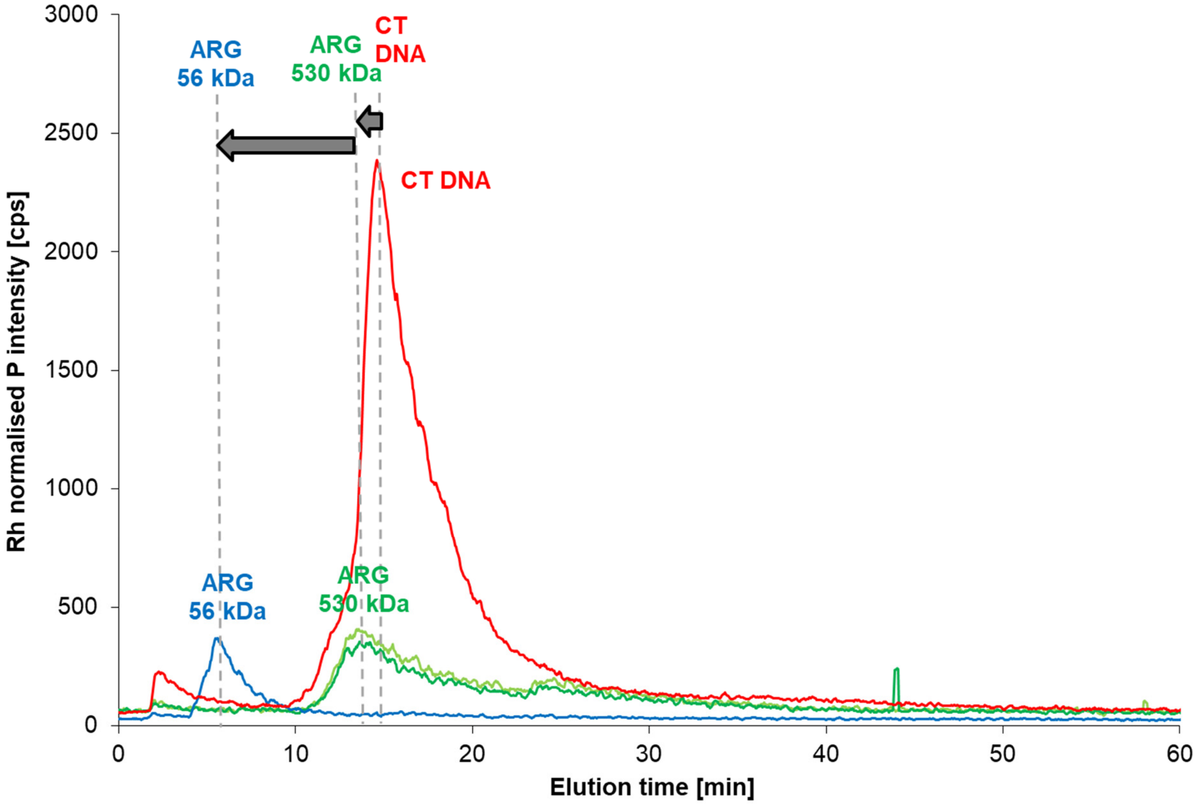

Supplement: Supplementary file 1 [file DataSheet1.PDF]
